# Histamine-induced biphasic activation of RhoA allows for persistent RhoA signaling

**DOI:** 10.1371/journal.pbio.3000866

**Published:** 2020-09-03

**Authors:** Jason Z. Zhang, Andy H. Nguyen, Shigeki Miyamoto, Joan Heller Brown, Andrew D. McCulloch, Jin Zhang

**Affiliations:** 1 Department of Bioengineering, University of California San Diego, La Jolla, California, United States of America; 2 Department of Pharmacology, University of California San Diego, La Jolla, California, United States of America; University of Michigan, UNITED STATES

## Abstract

The small GTPase RhoA is a central signaling enzyme that is involved in various cellular processes such as cytoskeletal dynamics, transcription, and cell cycle progression. Many signal transduction pathways activate RhoA—for instance, Gα_q_-coupled Histamine 1 Receptor signaling via Gα_q_-dependent activation of RhoGEFs such as p63. Although multiple upstream regulators of RhoA have been identified, the temporal regulation of RhoA and the coordination of different upstream components in its regulation have not been well characterized. In this study, live-cell measurement of RhoA activation revealed a biphasic increase of RhoA activity upon histamine stimulation. We showed that the first and second phase of RhoA activity are dependent on p63 and Ca^2+^/PKC, respectively, and further identified phosphorylation of serine 240 on p115 RhoGEF by PKC to be the mechanistic link between PKC and RhoA. Combined approaches of computational modeling and quantitative measurement revealed that the second phase of RhoA activation is insensitive to rapid turning off of the receptor and is required for maintaining RhoA-mediated transcription after the termination of the receptor signaling. Thus, two divergent pathways enable both rapid activation and persistent signaling in receptor-mediated RhoA signaling via intricate temporal regulation.

## Abstract 2

The small GTPase RhoA is a central signaling enzyme that is involved in various cellular processes such as cytoskeletal dynamics, transcription, and cell cycle progression. This study shows that histamine induces biphasic activation of RhoA via two divergent signaling pathways, allowing for intricate regulation of cellular processes.

## Introduction

The highly conserved small GTPase RhoA regulates various cellular processes such as cellular motility and transcription and is implicated in cancer [1]. RhoA regulates these processes by dynamically cycling between its active GTP-bound state and its inactive GDP-bound state. Activation of RhoA is regulated by guanine nucleotide exchange factors (GEFs) that facilitate the exchange of the bound GDP for GTP. RhoA has intrinsic GTPase activity to hydrolyze GTP to GDP, and GTPase activating proteins (GAPs) accelerate this hydrolysis by three orders of magnitude [2]. Guanine dissociation inhibitors (GDIs) further inhibit RhoA and preserve the GDP-bound RhoA state by inhibiting GDP dissociation. To ensure signaling specificity for achieving its many roles, RhoA is spatiotemporally regulated through these GTPase regulators, which are in turn specifically regulated. For instance, many RhoGEFs are activated by membrane recruitment [3,4] or by heterotrimeric G proteins [5,6], including Gα_q_-activatable p63 RhoGEF [7] and Gα_12/13_-activatable p115 RhoGEF [8].

Characterization and understanding of the temporal regulation of RhoA is important, as it has a huge impact on cellular processes [9,10]. In this study, we used a genetically encoded fluorescent biosensor DORA RhoA [3] to measure RhoA activation kinetics in single living cells and uncovered unique biphasic activation of RhoA stimulated by histamine. We then examined the molecular mechanisms and functional roles of the biphasic RhoA activation and discovered that the sustained second phase of RhoA activation is regulated by Ca^2+^-PKC-p115, can be decoupled from the receptor activity, and is required for maintaining RhoA-mediated transcription after termination of receptor signaling.

## Results

### Histamine induces biphasic activation of RhoA

**Fig 1:**
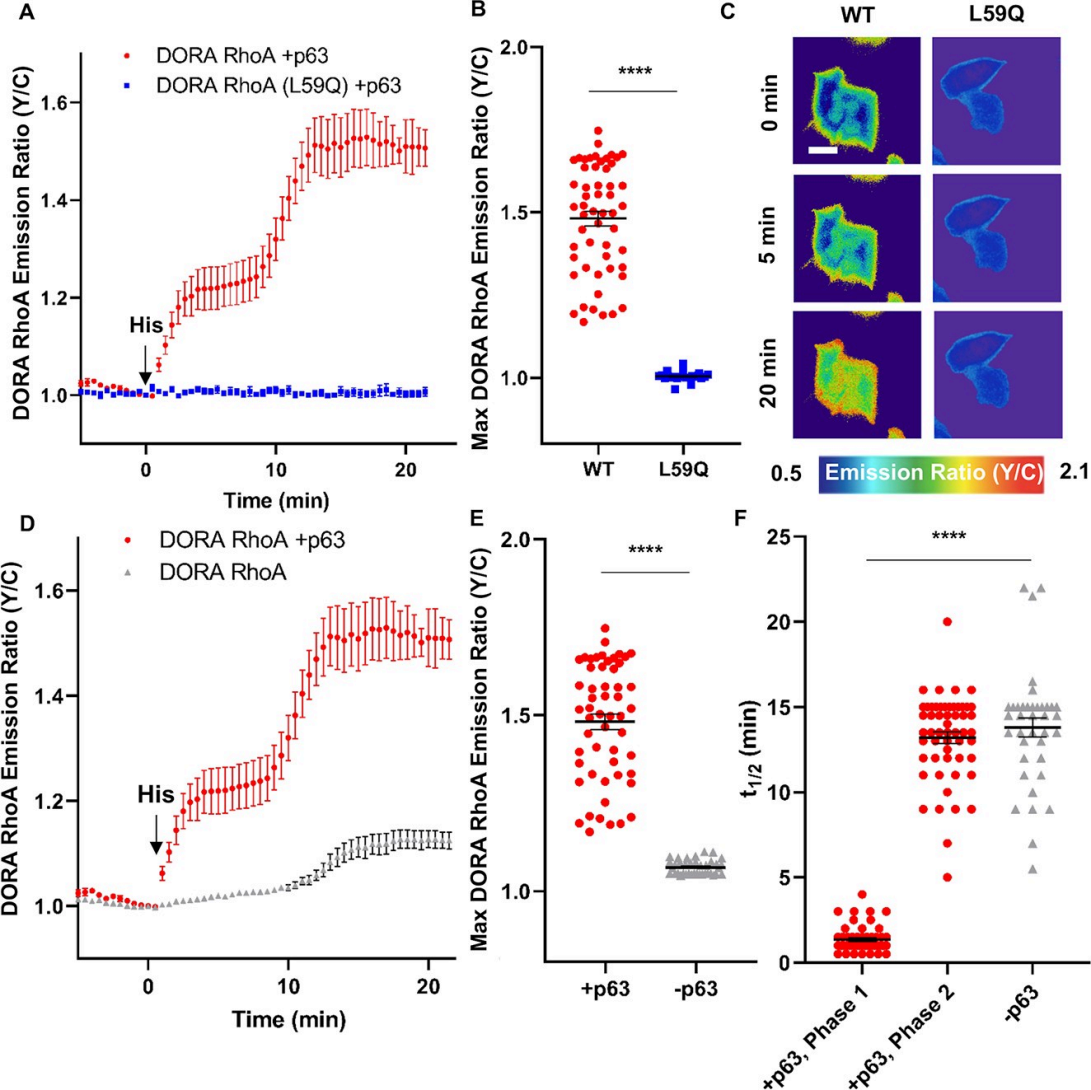
Histamine induces biphasic activation of RhoA. (**A-C**) Histamine (100 μM)–stimulated responses in HeLa cells coexpressing mCherry-tagged p63 and either DORA RhoA (red) or DORA RhoA (L59Q) (blue). (**A**) Representative average time courses ± SEM of Y/C emission ratio changes (DORA RhoA: *n* = 9 cells; DORA RhoA [L59Q]: *n* = 5 cells). Error bars indicate ± SEM. (**B**) Maximum (“Max”) emission ratio changes upon histamine (DORA RhoA: *n* = 54 cells; DORA RhoA [L59Q]: *n* = 23 cells). ****P < 0.0001; unpaired two-tailed Student’s *t* test. Bars indicated mean, error bars indicate ± SEM. (**C**) Pseudocolored images show the Y/C emission ratio in representative cells expressing the indicated constructs at 0, 5, and 20 min after histamine stimulation. (**D-F**) Histamine (100 μM)–stimulated DORA RhoA responses in HeLa cells transfected with DORA RhoA and p63 (red) or DORA RhoA alone (gray). (**D**) Representative average time courses ± SEM of Y/C emission ratio changes (DORA RhoA alone: *n* = 9 cells). (**E**) Maximum emission ratio changes upon histamine (DORA RhoA alone: *n* = 38 cells). ****P < 0.0001; unpaired two-tailed Student’s *t* test. (**F**) t_1/2_ after histamine stimulation for the first (t_1/2, Phase 1_) and second (t_1/2, Phase 2_) phases of the Y/C ratio increase in HeLa cells cotransfected with DORA RhoA and p63, and for the slow histamine-induced response (t_1/2_) in HeLa cells transfected with DORA RhoA only (DORA RhoA + p63: *n* = 54 cells; DORA RhoA alone: *n* = 38 cells). ****P < 0.0001; unpaired two-tailed Student’s *t* test. Scale bar, 10 μm. The underlying data for this figure can be found in S1 Data. t_1/2_, time to half-maximal responses; WT, wild type; Y/C, yellow/cyan.

To measure the dynamics of RhoA activation in response to histamine in cells, we used the Förster resonance energy transfer (FRET)-based DORA RhoA biosensor in HeLa cells expressing the canonical Gα_q_-activatable p63 RhoGEF (p63) [3,5,11]. The DORA RhoA sensor reports RhoA activation in single cells by converting changes in the RhoA nucleotide state to changes in FRET. This biosensor contains a FRET pair sandwiched between the Rho binding domain of Protein Kinase N1 (PKN1) and full-length RhoA. When RhoA is activated, the PKN1 domain binds to RhoA-GTP, inducing a conformational change and altering FRET, which is measured by an increase in the acceptor-to-donor (yellow/cyan [Y/C]) emission ratio [3]. To test the sensitivity of the DORA RhoA sensor to RhoGEFs and RhoGAPs, the sensor was coexpressed with either p63 RhoGEF, p115 RhoGEF, or p190 RhoGAP and the basal emission ratio was measured. Coexpression with p190 RhoGAP decreased the basal emission ratio by 18% whereas coexpression with either p63 RhoGEF or p115 RhoGEF increased the basal emission ratio by 20% and 25%, respectively, suggesting that the DORA RhoA sensor is sensitive to both RhoGEFs and RhoGAPs (S1A Fig). Upon stimulation with 100 μM histamine, HeLa cells expressing both p63 and DORA RhoA displayed a rapid increase in the Y/C emission ratio (22% ± 0.8% emission ratio increase, time to half-maximal response for Phase 1 [t_1/2,Phase 1_] = 1.4 ± 0.1 min, *n* = 54 [mean ± SEM; *n* = number of cells]) (Fig 1A–1C). This initial ratio increase was subsequently followed by an additional 26% ± 1.9% emission ratio increase (t_1/2,Phase 2_ = 13 ± 0.3 min) (Fig 1A–1C). Pretreatment with 100 μM H_1_ histamine receptor (H_1_HR) inverse agonist pyrilamine [12] abolished the histamine-induced response (S1B Fig). Control cells coexpressing p63 and DORA RhoA (L59Q), which contains an L59Q mutation in PKN1 to prevent RhoA binding [3], exhibited no detectable FRET changes in response to histamine stimulation (Fig 1A–1C), suggesting that the observed responses were specific. This histamine-induced biphasic activation of RhoA was also observed using another FRET-based RhoA sensor RhoA1G [13] (S1C Fig), suggesting that this biphasic behavior is a characteristic of RhoA and independent of biosensors. In the absence of p63 overexpression, DORA RhoA-expressing mouse embryonic fibroblast (MEF) cells, which endogenously express p63 RhoGEF [14], also displayed biphasic increases in DORA RhoA emission ratio, although the biphasic characteristics are not as prominent as in HeLa cells (S1D Fig). To further probe the temporal characteristics of RhoA activation in MEF cells, we performed Rhotekin pulldown experiments [15] in these cells. As shown in S1E Fig, histamine increased RhoA-GTP levels during two time periods (first period: 0–2 min, second period: 10–20 min). Although these two phases do not seem to be additive as shown by the biosensor data, possibly reflecting differences in the sensitivity and reversibility of the two assays, these data together suggest that histamine-induced biphasic activation of RhoA could occur with the endogenous level of p63. Finally, the biphasic response to Gα_q_-coupled receptor stimulation appears generalizable, as Clozapine N-Oxide (CNO) activation of the synthetic Gα_q_-coupled receptor (Gα_q_-DREADD) [16,17] also produced biphasic RhoA activation in HeLa cells coexpressing p63 (S1F Fig).

To explore whether these two phases of RhoA activation from histamine stimulation are both dependent on p63, we tested the histamine-induced RhoA response in HeLa cells lacking p63 overexpression. Interestingly, these cells exhibited a monophasic, slow increase in the DORA RhoA emission ratio (6.8% ± 3.3%, t_1/2_ = 14 ± 0.6 min, *n* = 38) upon histamine stimulation (Fig 1D–1F), the kinetics of which mirrored the second phase of the FRET response seen in p63-transfected cells (*P* = 0.38) (Fig 1D and 1F). These results suggest that histamine can induce biphasic increases in RhoA activity where the first phase appears to be dependent on p63. The amplitude of the response in the absence of p63 overexpression is lower than the second phase (26% ± 1.9%) of the biphasic RhoA activation when p63 was overexpressed, suggesting that p63 may also enhance the second phase of RhoA activation. We also explored the effect of RhoGAPs in our system by expressing p63 and p190 in HeLa cells, which still exhibited a biphasic increase in DORA RhoA emission ratio, suggesting that p190 does not play a major role in RhoA activation time scale and kinetics in our system (S1G Fig).

### Second phase of RhoA activation is dependent on the Ca^2+^/PKC/p115 signaling axis

**Fig 2:**
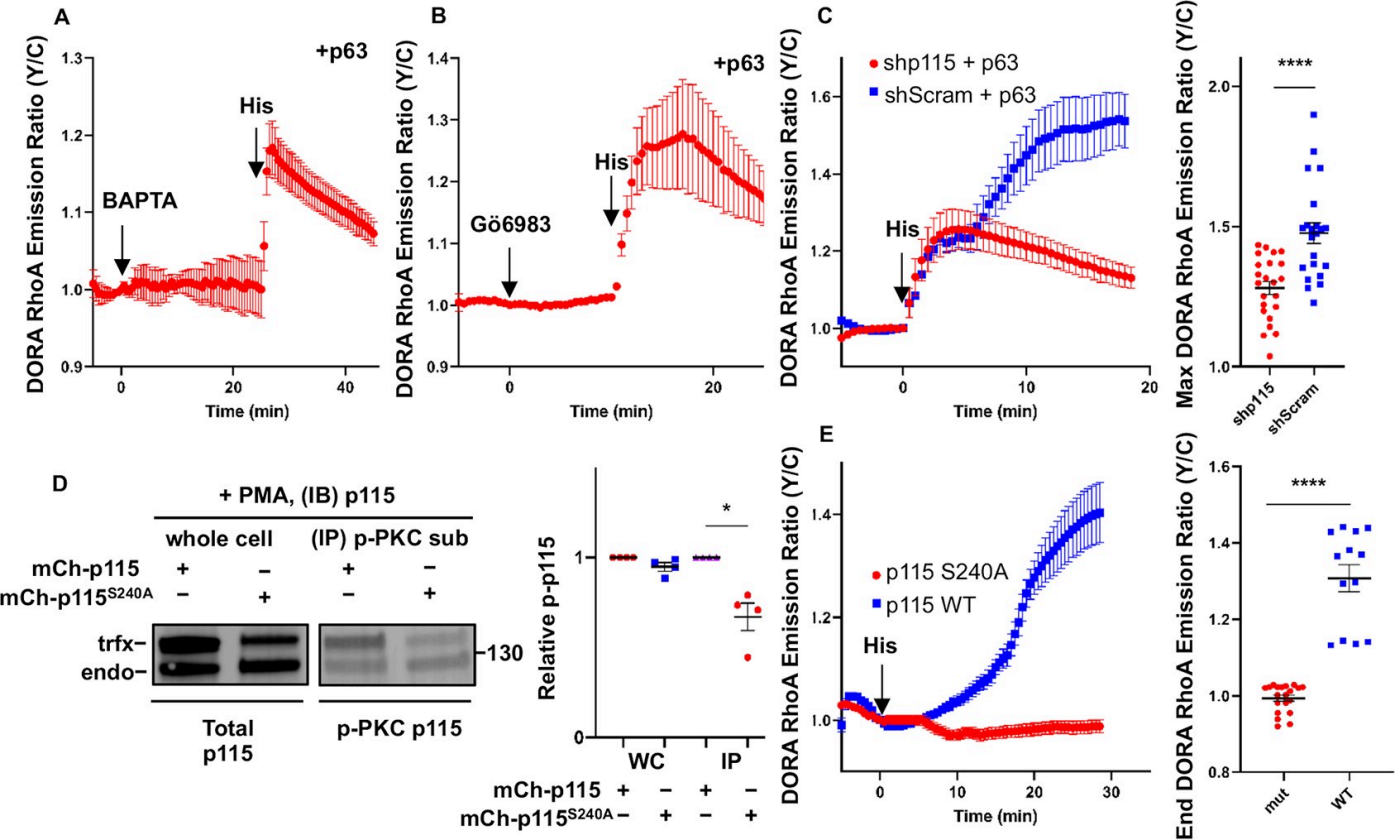
Second phase of RhoA activation is dependent on the Ca^2+^/PKC/p115 signaling axis. (**A-B**) Representative average time courses ± SEM of the Y/C emission ratio changes in HeLa cells coexpressing p63 and DORA RhoA. Cells were stimulated with either 20 μM BAPTA (**A**) (*n* = 4 cells) or stimulated with 1 μM Gö6983 (**B**) (*n* = 7 cells). Then cells were subsequently stimulated with 100 μM histamine. (**C**) Left: Representative average time courses ± SEM of the Y/C emission ratio changes in HeLa cells expressing DORA RhoA, p63, and either shRNA p115 (red) or shRNA Scrambled (blue) and stimulated with 100 μM histamine (shp115: *n* = 5 cells; shScrambled: *n* = 4 cells). Right: Maximum emission ratio changes upon histamine stimulation (shp115: *n* = 23 cells; shScrambled: *n* = 22 cells). ****P < 0.0001; unpaired two-tailed Student’s *t* test. (**D**) Left: Representative western blot images of HeLa cells show that PKC phosphorylates p115 on the serine 240 residue. HeLa cells expressing either mCherry-tagged p115 or p115 S240A were stimulated with 50 ng/mL PMA and subjected to immunoprecipitation with antibodies to phospho-PKC substrate. Whole-cell samples and the IP samples were immunoblotted for p115. Right: Densitometry analysis of the adjacent immunoblot is calculating the percentage of PKC-phosphorylated p115 over total p115 for the endogenous p115 (“endo,” lower band) and transfected p115 (“trfx,” upper band). Average percentage ± SEM shown in bar graph among the various transfection conditions (*n* = 4 for each condition). Endogenous p115 versus p115 S240A: *P* = 0.1; Transfected p115 versus p115 S240A: *P = 0.02; unpaired two-tailed Student’s *t* test. (**E**) Representative average time courses ± SEM of the Y/C emission ratio changes in DORA RhoA-expressing HeLa cells coexpressing either mCherry-tagged p115 (blue) or mCherry-tagged p115 S240A (red) and stimulated with 100 μM histamine (p115: *n* = 5 cells; p115 S240A: *n* = 12 cells). Right: Maximum emission ratio changes upon histamine stimulation (p115: *n* = 13 cells; p115 S240A: *n* = 20 cells). ****P < 0.0001; unpaired two-tailed Student’s *t* test. The underlying data for this figure can be found in S1 Data. BAPTA, 1,2-Bis(2-aminophenoxy)ethane-N,N,N′,N′-tetraacetic acid tetrakis (acetoxymethyl ester); Gö6983, 3-[1-[3-(Dimethylamino)propyl]-5-methoxy-1H-indol-3-yl]-4-(1H-indol-3-yl)-1H-pyrrole-2,5-dione; IB, immunoblotting; IP, immunoprecipitated; PMA, phorbol myristate acetate; WC, whole cell lysate; WT, wild type; Y/C, yellow/cyan.

Like all Gα_q_-coupled receptors, stimulation of the H_1_HR increases intracellular Ca^2+^ levels and PKC activity; thus, we next probed the role of Ca^2+^. In HeLa cells transfected with p63 and DORA RhoA and pretreated with 20 μM of the intracellular calcium chelator 1,2-Bis(2-aminophenoxy)ethane-N,N,N′,N′-tetraacetic acid tetrakis (acetoxymethyl ester) (BAPTA), histamine increased the emission ratio rapidly by 13% ± 2.2% (t_1/2_ = 0.7 ± 0.09 min, *n* = 18) with no subsequent second increase in emission ratio (Fig 2A). Addition of BAPTA 5 min after histamine stimulation also eliminated the second increase in DORA RhoA emission ratio (S2A Fig). In addition, BAPTA pretreatment eliminated the delayed increase but not the immediate increase in RhoA1G emission ratio upon histamine stimulation (S3A Fig), suggesting that the observed effect of chelating intracellular calcium on RhoA kinetics is independent of the RhoA sensor. Furthermore, in p63-expressing cells imaged in Ca^2+^ free media containing 1 mM Ethylene glycol-bis(β-aminoethyl ether)-N,N,N′,N′-tetraacetic acid (EGTA) to eliminate extracellular calcium, histamine stimulation again increased the DORA RhoA emission ratio rapidly by 14% ± 2.3% (t_1/2_ = 0.8 ± 0.05 min, *n* = 18) with no subsequent increase (S2B Fig), suggesting that removal of Ca^2+^ abolishes the slow phase of RhoA activation from histamine. In contrast, increasing intracellular Ca^2+^ by addition of 1 μM ionomycin in p63-expressing cells led to a gradual increase the DORA RhoA emission ratio by 12% ± 2.6% (*n* = 12). Subsequent histamine stimulation again rapidly increased the emission ratio by an additional 30% ± 3.9% (t_1/2_ = 1.2 ± 0.2 min, *n* = 12) (S2C Fig). These data suggest that the second phase of RhoA activation is dependent on Ca^2+^ and an increase in intracellular Ca^2+^ alone is sufficient to activate RhoA.

Given that PKC is activated by Ca^2+^ and has been implicated in activation of RhoA [18,19], we next assessed PKC’s role in histamine-induced RhoA activation. When PKC is inhibited by 1 μM 3-[1-[3-(Dimethylamino)propyl]-5-methoxy-1H-indol-3-yl]-4-(1H-indol-3-yl)-1H-pyrrole-2,5-dione (Gö6983), subsequent histamine stimulation of p63-expressing cells rapidly (t_1/2_ = 0.9 ± 0.2 min) increased the DORA RhoA emission ratio by 34% ± 3.0% (*n* = 16) with no subsequent increase in emission ratio (Fig 2B), similar to what was observed in the BAPTA experiments. Addition of Gö6983 5 min after histamine stimulation also eliminated the second increase in DORA RhoA emission ratio (S2D Fig). In addition, Gö6983 pretreatment eliminated the delayed increase but not the immediate increase in RhoA1G emission ratio upon histamine stimulation (S3B Fig), suggesting that the observed effect of PKC inhibition on RhoA kinetics is independent of the RhoA sensor. On the other hand, activation of PKC by 50 ng/mL phorbol myristate acetate (PMA) increased the DORA RhoA emission ratio of p63-expressing cells by 12% ± 2.6% (*n* = 13). Subsequent histamine stimulation rapidly increased the DORA RhoA emission ratio by an additional 13% ± 2.8% (t_1/2_ = 0.6 ± 0.05 min, *n* = 13) (S2E Fig). Even when p63 was not expressed in cells, PMA treatment can lead to a gradual increase of the DORA RhoA emission ratio by 7.2% ± 0.8% (*n* = 15), suggesting that PKC can activate RhoA independent of p63. Subsequent PKC antagonism by Gö6983 decreased the DORA RhoA emission ratio to near pre-PMA stimulation emission ratio values (−4.7% ± 0.8%, *n* = 15) (S2F Fig). Overall, our data suggests that the second phase of RhoA activation induced by histamine is dependent on PKC and PKC activation alone is sufficient to activate RhoA.

Next, we set out to identify the RhoGEF involved in Ca^2+^/PKC-mediated activation of RhoA. Among the potential links between PKC and RhoA, several studies report that PKC can increase RhoA activity by phosphorylating the Gα_12/13_-activatable p115 RhoGEF (p115) [18,20]. To test the role of p115 in our system, we knocked down endogenous p115 in HeLa cells (S2G Fig) and measured the histamine-induced RhoA activation. In cells expressing p115 shRNA and p63, histamine addition rapidly increased the DORA RhoA emission ratio (28% ± 2.4%, t_1/2_ = 1.2 ± 0.1 min, *n* = 23) with no subsequent increase in emission ratio (Fig 2C). In contrast, cells with the scrambled shRNA control and p63 and stimulated with histamine displayed a biphasic increase (48% ± 3.6%, *P* < 0.0001) in the DORA RhoA emission ratio (t_1/2, Phase 1_ = 1.9 ± 0.3 min, t_1/2, Phase 2_ = 16 ± 1.5 min, *n* = 22) (Fig 2C), similar to the histamine-induced RhoA activation in cells with p63 and no shRNA (Fig 1A). In the absence of p63, histamine induced no increase in DORA RhoA emission ratio in the p115 shRNA-expressing cells (S2H Fig), in contrast to the delayed increase in the scrambled shRNA control (S2I Fig). When p115 was coexpressed with p63, the biphasic activation of RhoA from histamine stimulation was still present and the maximum DORA RhoA emission ratio was higher (80% ± 2.9%) compared to when only p63 was expressed (49% ± 3.4%, *P* < 0.0001) (S2J Fig). Moreover, this increase in DORA RhoA emission ratio in the p115 and p63 coexpression case is primarily contributed by the second phase of RhoA activation (S2K Fig). Together, these data suggest that p115 RhoGEF is required for the second phase of RhoA activation from histamine.

### PKC phosphorylation on serine 240 as the critical link between PKC and RhoA

Although several studies show that thrombin or tumor necrosis factor (TNFα) stimulation induces PKCα-mediated phosphorylation and activation of p115 to regulate endothelial cell permeability, the specific phosphorylation site has not yet been determined, and it is not clear if this PKC dependent mechanism [18,20] occurs downstream of Gα_q_. To determine if p115 is phosphorylated by PKC in response to histamine, we immunoprecipitated p115 from cells treated with PKC activator and/or inhibitor and examined its phosphorylation with an anti-phospho-PKC substrate antibody. PMA treatment increased PKC phosphorylation of p115 by 2-fold (2.1 ± 0.4, *n* = 5) compared with no drug treatment (*P =* 0.02). Inhibition of PKC with Gö6983 abolished PMA-induced phosphorylation (1.1 ± 0.1, *n* = 4) (S4A Fig and S4B Fig).

Serine 240 was predicted to be a PKC phosphorylation site based on Kinexus PhosphoNet [21] predictions using kinase consensus motif information (S4C Fig). We therefore mutated this serine 240 to an alanine (p115 S240A), tagged it with mCherry, and tested its phosphorylation by PKC. Because of technical reasons (see Materials and methods), we modified the protocols for examining the phosphorylation of mCherry-tagged p115. We treated cells expressing either mCherry-tagged wild-type p115 or S240A mutated p115 with PMA to activate PKC, immunoprecipitated phosphorylated PKC substrates [22], and probed for p115. The amount of PKC-phosphorylated endogenous p115 (lower band) was similar between the two samples, whereas PKC phosphorylation of the mCherry-tagged p115 S240A (upper band) decreased by 1.6-fold (1.6 ± 0.2, *n* = 4) compared to mCherry-tagged wild-type p115 (Fig 2D), suggesting that PKC phosphorylation of p115 is at least partly through this site.

To test whether serine 240 plays a key role in PKC-mediated RhoA activation, we examined DORA RhoA responses in cells expressing either wild-type or S240A mutant of p115. Histamine stimulation led to a 31% ± 3.5% increase in DORA RhoA emission ratio in cells overexpressing wild-type p115 (p115 protein expression is approximately 2× increased based on Fig 2D), with kinetics consistent with the second phase (t_1/2_ = 11 ± 1.8 min, *n* = 13) (Fig 2E). In contrast, histamine induced no change in DORA RhoA emission ratio in the presence of p115 S240A (0.01% ± 0.8%, *n =* 20) (Fig 2E), suggesting that not only did the S240A mutation abolish the PKC-mediated RhoA activation but also p115 S240A exhibited some dominant negative effect. Similar results were seen in cells stimulated with PMA, as p115 S240A abolished PMA-induced RhoA activation (S4D Fig). Whereas membrane recruitment of p115 RhoGEF (most commonly through activation of Gα_12/13_-coupled receptors) increases RhoA activity [23–29], neither histamine nor the histamine receptor antagonist pyrilamine affected p115 localization (S4E Fig), suggesting that histamine-induced PKC phosphorylation of p115 activates p115 in a noncanonical way that is independent of acute membrane recruitment. Overall, these data suggest that PKC phosphorylates serine 240 on p115 to activate its RhoGEF activity, which is responsible for the second phase of histamine-induced RhoA activation. These results also uncovered a critical mechanistic link between PKC and RhoA and provided the molecular mechanism underlying a noncanonical signaling axis that connects Gα_q_-coupled receptors to RhoA.

### The Ca^2+^/PKC/p115 signaling axis enables persistent RhoA signaling

**Fig 3:**
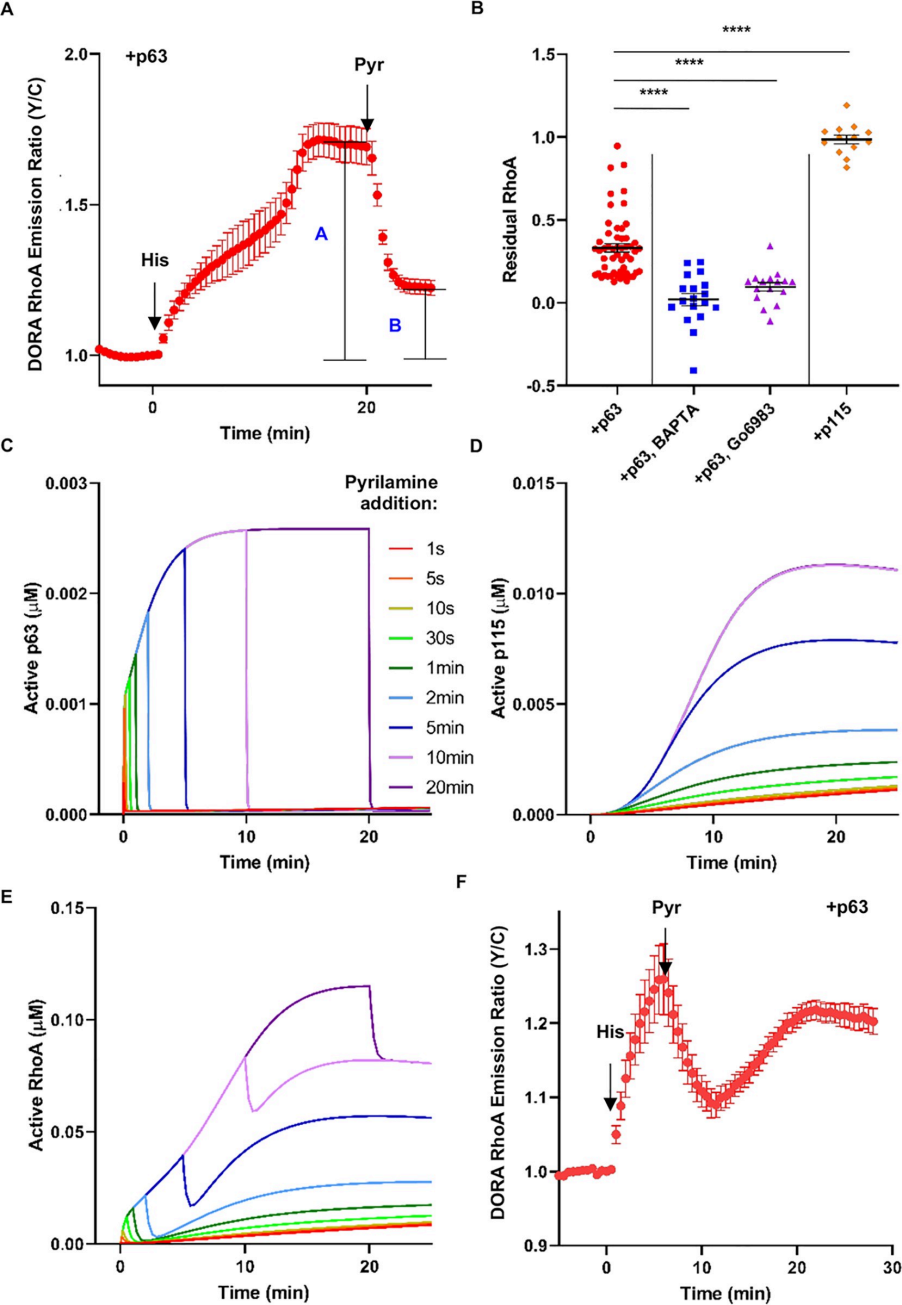
The Ca^2+^/PKC/p115 signaling axis enables persistent RhoA signaling. (**A**) Residual RhoA activity is defined as the DORA RhoA Y/C emission ratio after addition (B) divided by the maximum increase of the DORA RhoA Y/C emission ratio after histamine stimulation (A). (**B**) Residual RhoA activity for various conditions to either increase (+p115, *n* = 13 cells) or decrease (+p63 with either BAPTA [*n* = 18 cells] or Gö6983 pretreatment [*n* = 16 cells]) flux into the Ca^2+^/PKC/p115 signaling axis. Compared to the residual RhoA activity in cells expressing p63 (+p63, *n* = 54 cells) with no drug pretreatment, conditions with increased flux into the Ca^2+^/PKC/p115 signaling axis also increased residual RhoA activity. For all comparisons to +p63 condition: ****P < 0.0001; ordinary one-way ANOVA followed by Dunnett’s multiple-comparisons test (versus +p63). (**C-E**) Computational simulations of adding pyrilamine at different time points after histamine stimulation and measuring concentrations of active p63 (**C**), active p115 (**D**), or active RhoA (**E**). (**F)** Average time course from multiple experiments ± SEM of the Y/C emission ratio changes in HeLa cells expressing DORA RhoA and p63. Cells were stimulated with 100 μM histamine, and then 100 μM pyrilamine was added 5.5 min afterwards (*n* = 17 cells). The underlying data for this figure can be found in S1 Data. BAPTA, 1,2-Bis(2-aminophenoxy)ethane-N,N,N′,N′-tetraacetic acid tetrakis (acetoxymethyl ester); Gö6983, 3-[1-[3-(Dimethylamino)propyl]-5-methoxy-1H-indol-3-yl]-4-(1H-indol-3-yl)-1H-pyrrole-2,5-dione; Y/C, yellow/cyan.

Given that stimulation of the histamine receptor activates RhoA in a biphasic manner via p63-dependent and p115-dependent pathways, we wondered whether active receptors are required to maintain the activation of RhoA. We tested this experimentally by applying the H_1_HR inverse agonist pyrilamine after the biphasic activation reaches a plateau. As a metric for measuring the RhoA activity after receptor inactivation, we devised the residual RhoA activity metric, which is the ratio of the DORA RhoA emission ratio post pyrilamine over the emission ratio post histamine (Fig 3A and 3B, see Materials and methods). In biphasic-responding cells overexpressing p63 (with endogenous p115), pyrilamine addition decreased the RhoA activity by 67%, leaving 33% ± 2.6% as residual RhoA activity (*n* = 54) (Fig 3A and 3B). In cells where flux into the Ca^2+^/PKC/p115 signaling axis is largely reduced, such as p63-expressing cells pretreated with either BAPTA or Gö6983, the residual RhoA activity was largely eliminated (BAPTA: residual RhoA activity = 2% ± 3.7%, *n* = 18; Gö6983: residual RhoA activity = 9.7% ± 2.6%, *n* = 16) (Fig 3B). In contrast, cells overexpressing p115 in the absence of p63 exhibited no decrease when pyrilamine was added post histamine stimulation (residual RhoA activity = 99% ± 2.6%, *n* = 13) (Fig 3B). Testing various other conditions that either increase or decrease flux into the Ca^2+^/PKC/p115 signaling axis showed a consistent trend that increasing Ca^2+^/PKC/p115 signaling also increased residual RhoA activity (S5A Fig). Furthermore, PKC inhibition by Gö6983 after pyrilamine addition completely reversed the DORA RhoA emission ratio back down to basal levels (residual RhoA activity = 4.3% ± 2.9%, *n* = 19) (S5B Fig), suggesting that PKC remains active and plays an important role in maintaining the residual RhoA activity. Indeed, whereas histamine induces a transient increase in Ca^2+^ (S6A Fig), both PKC activity as detected by ExRai CKAR [30] (S6B Fig) and phosphorylation of p115 (S6C Fig) remain elevated even after pyrilamine treatment. Conceptually, these data suggest that the Ca^2+^/PKC/p115 signaling pathway enables persistent RhoA signaling where previous histamine-induced RhoA activation is retained even after histamine receptor inactivation.

The pyrilamine experiments gave hints into the differential regulation of the p63 and p115-dependent pathways by H_1_HR, and we constructed a computational model to more directly probe the impact of the receptor state on RhoA activity. By modeling binding events with mass action kinetics and enzyme-mediated events with Michaelis-Menten kinetics, the model captured the characteristics of the biphasic RhoA activation (t_1/2,Phase 1_ = 1.9 min, t_1/2,Phase 2_ = 8.6 min) when both p63 and p115 are present (S7 Fig). In addition, monophasic histamine activation is fast (t_1/2_ = 1.4 min) if only p63 is present and slow (t_1/2_ = 9.4 min) if only p115 is present (S7B Fig and S7C Fig). In alignment with experimental data, the model predicts that upon pyrilamine addition, RhoA activity goes down to prestimulation levels when only p63 is present, decreases by 35% when both p63 and p115 are present, and has no effect when only p115 is present in the simulation (S7B Fig).

To further probe the impact of the H_1_HR state on RhoA activity, receptor inactivation by pyrilamine over a range of times post histamine stimulation was simulated, and the amount of active p63, active p115, and active RhoA was plotted (Fig 3C–3E). Whereas p63 activity was immediately turned off by pyrilamine treatment (Fig 3C) after histamine stimulation, p115 activity can still accumulate even if pyrilamine was added soon after histamine stimulation (Fig 3D). The overall RhoA activation kinetics and strength were altered depending on the duration of receptor activation. However, regardless of when pyrilamine was added after histamine stimulation and how transiently the receptor is activated, RhoA activity gradually increases after a transient decrease (Fig 3E), a prediction that was validated experimentally (Fig 3F). The computational predictions and experimental data suggest that the first, p63-dependent phase of RhoA activation requires continuously active receptor whereas the second, p115-depedent phase can be decoupled from the receptor activity, allowing for this persistent RhoA signaling.

### Persistent RhoA signaling is important for transcriptional activity following transient receptor activation

**Fig 4:**
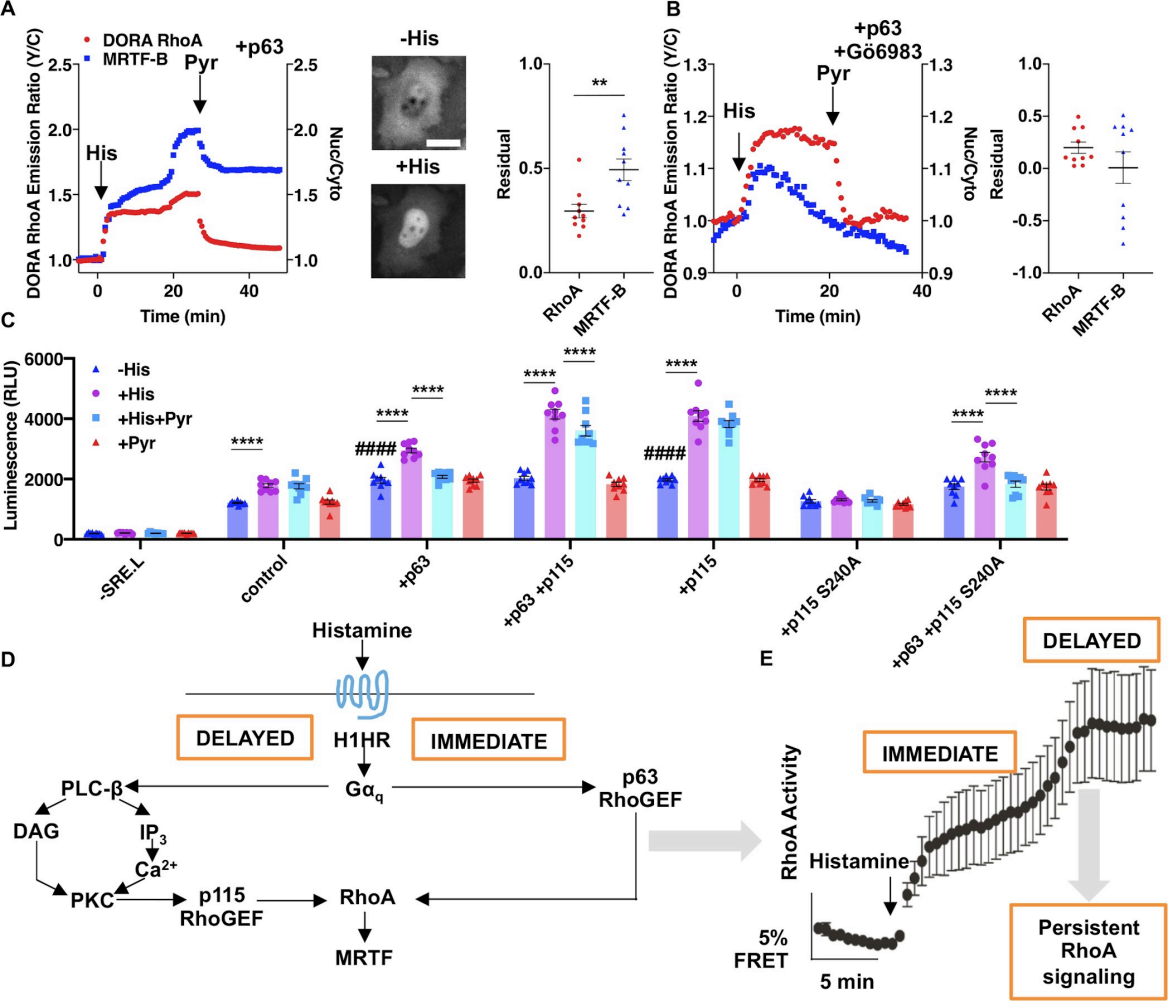
Persistent RhoA signaling is important for transcriptional activity following transient receptor inactivation. (**A, B**) RhoA activation and MRTF-B nuclear translocation in HeLa cells expressing DORA RhoA, p63, and mTagBFP2-tagged MRTF-B stimulated with 100 μM histamine and then 100 μM pyrilamine, without (**A**) and with (**B**) Gö6983 pretreatment. (**A**) Left: Representative time course of biphasic-responding HeLa cells. DORA RhoA Y/C emission ratio changes on left axis and nuclear to cytosol ratio (“Nuc/Cyto”) of MRTF-B on right axis. Middle: Representative BFP epifluorescence images of HeLa cells expressing mTagBFP2-tagged MRTF-B before and after histamine stimulation. Right: Residual RhoA activity and residual MRTF-B in the nucleus (*n* = 10 cells for each metric). Scale bar, 10 μm. (**B**) Left: Representative time course of monophasic-responding HeLa cells. Right: Residual RhoA activity and residual MRTF-B in the nucleus (*n* = 10 cells for each metric). (**C**) Average bioluminescence in cells with various transfection and drug stimulation conditions (*n* = 6 wells for each condition). Number signs are in comparison to control–His condition; asterisks are comparisons that are indicated by horizontal lines: ****P < 0.0001, ####*P* < 0.0001; unpaired two-tailed Student’s *t* test. (**D**) Summary of findings. Histamine binding to the Gα_q_-coupled H_1_HR activates the Gα_q_-activatable p63 RhoGEF to immediately activate RhoA. H_1_HR activation also leads to the canonical Gα_q_ pathway where intracellular Ca^2+^ levels and PKC activities are increased. PKC phosphorylates p115 RhoGEF on the serine 240 residue, which in turn activates p115 to activate RhoA during the delayed phase. Activation of RhoA leads to nuclear translocation of MRTF-A/B to increase transcriptionally activity. (**E**) The two phases of RhoA activation leads to different phenotypic responses, in which the delayed phase leads to persistent RhoA signaling and transcriptional activity. The underlying data for this figure can be found in S1 Data. BFP, blue fluorescent protein; DAG, diacylglycerol; FRET, Förster resonance energy transfer emission ratio; GEF, guanine nucleotide exchange factor; Gö6983, 3-[1-[3-(Dimethylamino)propyl]-5-methoxy-1H-indol-3-yl]-4-(1H-indol-3-yl)-1H-pyrrole-2,5-dione; H_1_HR, H_1_ histamine receptor; IP, immunoprecipitated; MRTF, myocardin-related transcription factor; RLU, relative light unit; Y/C, yellow cyan.

To explore the functional role of this p115-dependent persistent RhoA signaling, we examined the downstream effects of RhoA activation. RhoA activates various transcription factors such as inducing the translocation of myocardin-related transcription factor (MRTF-A/B) into the nucleus [1]. While in the nucleus, MRTF interacts with Serum Response Factor (SRF) to activate transcription of target genes [31]. Thus, to monitor RhoA-mediated transcriptional events, we first measured the nuclear localization of MRTF [32–34]. In p63-expressing cells that exhibited histamine-induced biphasic RhoA activation, nuclear translocation of MRTF-B was also biphasic (Fig 4A). In these cells, subsequent pyrilamine addition decreased RhoA activity and MRTF-B nuclear localization but not to prestimulation levels. Interestingly, the decrease in MRTF-B nuclear localization is smaller than the decrease in RhoA activity, suggesting that the persistent RhoA signaling is amplified downstream of RhoA (RhoA: residual RhoA activity = 30% ± 3.2%, *n* = 10; MRTF-B: residual MRTF-B nuclear localization = 49% ± 5.2%, *n* = 10, *P* = 0.0056) (Fig 4A and S8A Fig). In contrast, Gö6983-pretreated cells expressing p63 exhibited fast, monophasic RhoA activation and relatively transient MRTF-B nuclear translocation (Fig 4B and S8B Fig). In these monophasically responding cells, the transient MRTF-B nuclear translocation almost completely reversed to prestimulation levels. In addition, pyrilamine had a small effect to further reduce nuclear localization of MRTF-B, and after pyrilamine treatment there was essentially no residual nuclear localization (RhoA: residual RhoA activity = 20% ± 5.4%, *n* = 10 cells; MRTF-B: residual MRTF-B nuclear localization = 0.8% ± 15%, *n* = 10 cells, *P* = 0.3) (Fig 4B and S8B Fig). In cells where biphasic RhoA activation occurred, residual MRTF-A nuclear localization was also observed after pyrilamine addition (S9 Fig). These data suggest that the biphasic behavior of RhoA activation impacts MRTF-B and MRTF-A nuclear translocation kinetics and retention of nuclear localization after histamine receptor inactivation.

Looking further downstream, we measured MRTF/SRF-mediated transcription via the SRE.L luciferase reporter [35]. Basal RhoA-mediated transcriptional activity was increased compared to the endogenous condition (SRE.L) when wild-type p63 or p115 RhoGEFs were overexpressed (“–His” comparison marked by number signs in Fig 4C of control to either +p63 or +p115 conditions: *P* < 0.0001). Histamine stimulation (30 min) increased RhoA-mediated transcription, which was measured 24 h later, when either wild-type p63 or p115 was overexpressed; the only case in which histamine had no effect on RhoA-mediated transcription was when only the PKC phosphorylation–defective mutant of p115 (SRE.L + p115 [S240A]) was expressed (Fig 4C), consistent with the data showing that histamine does not activate RhoA when p115 S240A was expressed (Fig 2E). Pyrilamine incubation (30 min) after histamine stimulation (30 min) (+His +Pyr) decreased RhoA-mediated transcription compared to histamine-alone stimulation (+His) when p63 was expressed, whereas expression of wild-type p115 attenuated pyrilamine’s effect in decreasing RhoA-mediated transcription (Fig 4C). For instance, expression of only wild-type p115 (+ p115) abrogated the difference in RhoA-mediated transcription between histamine alone (+His) versus histamine and then pyrilamine (+His +Pyr) (Fig 4C) (+ p115 condition: +His, relative light units [RLU] = 4,084 ± 175; +His +Pyr, RLU = 3,823 ± 120, *n* = 9, *P* = 0.15). These data suggest that even though both p63 and p115 play a role in both basal and histamine-induced increases in RhoA-mediated transcription, p115 plays a unique role in transducing persistent RhoA signaling into sustained transcriptional activity following transient receptor activation.

## Discussion

By measuring signaling kinetics through FRET-based biosensors, we discovered that stimulation of Gα_q_-coupled receptors such as the histamine receptor induces biphasic RhoA activation. We determined the mechanisms underlying these two activation phases to be attributed to two pathways that bifurcate at Gα_q_ (Fig 4D). The immediate phase of RhoA activation is by Gα_q_ activating p63 RhoGEF, which in turn activates RhoA. The delayed phase is through the canonical Gα_q_ pathway to increase Ca^2+^ levels and PKC activity. Following activation of PKC by diacylglycerol (DAG) and Ca^2+^, p115 RhoGEF is activated through PKC-mediated phosphorylation of serine 240, leading to further activation of RhoA. Furthermore, the immediate phase is tightly regulated by the histamine receptor while the delayed phase is decoupled from the receptor after initial activation, leading to persistent RhoA signaling. This persistent RhoA signaling is also amplified and transduced into sustained transcriptional activity that is uncoupled from the activation state of the receptor (Fig 4E). Our data suggest that this observed persistence is not encoded at the RhoA level but more upstream. Histamine-induced Ca^2+^ increases allow for Ca^2+^ binding and membrane recruitment of PKC [36]. Although Ca^2+^ increases are transient (S6A Fig), which would affect PKC activity, our computational model predicts that DAG levels are sustained even after pyrilamine addition, which allows for persistent PKC activity even after receptor inactivation. This persistent PKC activity and relatively slow phosphatase activity (S6B Fig and S6C Fig) allow the sustained phosphorylation of p115 RhoGEF. Altogether, we postulate that the mechanism for the observed persistent RhoA signaling is through continuous PKC activity from sustained DAG levels and slow dephosphorylation on p115 RhoGEF. These findings are likely a general feature of RhoA signaling [37], as biphasic RhoA activation is not only seen by histamine stimulation but also other Gα_q_-coupled receptors such as the synthetic Gα_q_-DREADD (S1F Fig).

To reveal the impact of the upstream signaling pathways on RhoA kinetics, we combined computational modeling with live-cell fluorescence imaging. By measuring spatiotemporal signaling dynamics at single-cell resolution using fluorescent biosensors, we can obtain quantitative kinetic information and then incorporate these measurements to form a biologically accurate and relevant model. Although many studies have applied computational models to answer various questions in the signaling field [38–40], only a small number of studies have incorporated quantitative biosensor imaging to revise and validate their computational models [41–46]. Here, we computationally simulated the pathways responsible for RhoA activation to tease apart the impact of the histamine receptor state on RhoGEF and RhoA activity. Whereas the FRET-based biosensors measured RhoA activity one condition at a time, the computational modeling allowed us to test many conditions quickly and evaluate metrics that cannot be measured with current tools, such as the activity state of specific RhoGEFs. Modeling predictions were then tested experimentally. Combining both computational and experimental approaches, we concluded that the PKC/p115 pathway is responsible for maintaining RhoA signaling after receptor inactivation. In the future, the computational model can be further expanded to address the role of other players in these complex pathways, such as protein phosphatases in regulating RhoA kinetics.

In summary, our study identified a set of biochemical events to produce biphasic activation of RhoA from histamine stimulation. These two phases were regulated by the receptor differently and impacted transcription with different kinetics to allow both rapid kinetics and persistent signaling. These studies allow for greater appreciation of the intricate organization of RhoA signaling [37], providing mechanisms underlying RhoA signaling specificity.

## Materials and methods

### Plasmid construction

All assembly of constructs was performed using Gibson assembly (NEB 2x High-Fidelity Master Mix). mCherry-tagged p63 RhoGEF, DORA RhoA, and DORA RhoA (L59Q) were constructed previously [3]. To make mCherry-tagged p115 RhoGEF, Gibson assembly of PCR products (Q5 High-Fidelity Kit, New England BioLabs) amplified from mCh-p63 for mCherry using the forward primer (lowercase letters are Gibson assembly overhangs and uppercase letters are priming regions) 5′-cactatagggagacccgccaccATGGTGAGCAAGGGCGAGGA-3′ and reverse primer 5′-GCATGGACGAGCTGTACAAG-3′ and from pCEFL-p115 plasmid (gift of Silvio Gutkind, University of California San Diego, CA) for p115 using forward primer 5′-gcatggacgagctgtacaagATGGAAGACTTCGCCCGAG-3′ and reverse primer 5′-GCCTGGCTGCACTTGAgaattctgcagatatccagc-3′. Assembly of S240A mutant of p115 was generated via Gibson assembly of PCR products that introduced the mutation in mCh-p115 using the forward primer 5′-aagaaggcaggtagaaatTTCTTCCGGAAAAAGGTGATG-3′ and the reverse primer 5′-gaagaaatttctacctgcCTTCTTGTCTCCACTCTTGGTC-3′. shRNA p115 and Scrambled were generated from Santa Cruz Biotech (sc-48363). Generation of mTagBFP2-tagged MRTF-A was through Gibson assembly of PCR products amplified from p3xFLAG-MKL1 (gift from Ron Prywes [Addgene plasmid # 11978; http://n2t.net/addgene:11978; RRID:Addgene_11978)] for MRTF-A using the forward primer 5′- aactggggcacaagcttaatggaggtactggtggaagtATGCCGCCTTTGAAAAGTCC-3′ and the reverse primer 5′-taaacgggccctctagactaCTACAAGCAGGAATCCCAGTG-3′ and from mTagBFP2-pBAD (gift from Michael Davidson [Addgene plasmid # 54572; http://n2t.net/addgene:54572; RRID:Addgene_54572]) for mTagBFP2 using the forward primer 5′-agacccaagctggctagcgtttaaacttaagcttgggccaccATGAGCGAGCTGATTAAGGAG-3′ and the reverse primer 5′-ACTGGGGCACAAGCTTAAT-3′. Generation of mTagBFP2-tagged MRTF-B was similar to MRTF-A but using pmVenus-C2-MmMKL2 (gift from Dorus Gadella [Addgene plasmid # 67894; http://n2t.net/addgene:67894; RRID:Addgene_67894]) for MRTF-B using the forward primer 5′-aactggggcacaagcttaatggaggtactggtggaagtATGATCGATAGCTCCAAGAAGC-3′ and the reverse primer 5′-GTTTAAACGGGCCCTCTAGACTAgtcccatggcagcg-3′. The RhoA1G plasmid was a gift from Klaus Hahn (Addgene plasmid #12150; http://n2t.net/addgene:12150; RRID:Addgene_12150). The pKH3-p190 GAP plasmid was a gift from Ian Macara (Addgene plasmid #15547; http://n2t.net/addgene:15547; RRID:Addgene_15547).

### Cell culture and transfection

HeLa cells were cultured in Dulbecco modified Eagle medium (DMEM; Gibco) containing 1 g/L glucose and supplemented with 10% (v/v) fetal bovine serum (FBS, Sigma) and 1% (v/v) penicillin-streptomycin (Pen-Strep, Sigma-Aldrich). All cells were maintained in a humidified incubator at 37°C with a 5% CO_2_ atmosphere. Prior to transfection, cells were plated onto sterile 35-mm glass-bottomed dishes and grown to 50%–70% confluence. Cells were then transfected using Lipofectamine 2000 (Invitrogen) or Calcium Phosphate (for studies that involve shRNA) and grown an additional 24 h before imaging.

### Bioluminescence assay

HeLa cells seeded onto a 6-well plate (Falcon) were transfected with SRE.L and indicated plasmids. Twenty-four hours after transfection, cells were passaged onto a 96-well plate (Corning Costar) in triplicates. If indicated, cells were stimulated with either histamine for 30 min, pyrilamine for 30 min, or histamine for 30 min and then pyrilamine for 30 min. After drug addition, drug was washed away at least three times with fresh media. Twenty-four hours later, media was replaced with PBS and 150 μg/mL D-luciferin (Gold Biotechnology) was added to cells and bioluminescence was measured on the Tecan Spark 20M.

### Immunoprecipitation and western blot

HeLa cells were washed 3× with 37°C Hank’s balanced salt solution (HBSS) and then indicated drugs were added for 30 min in 37°C non-CO_2_ incubator. HeLa cells were subsequently lysed (1 mM NaF, 1 mM Na_3_VO_4_, 10 nM Calyculin A, 1 mM PMSF, 1 mg Complete, EDTA-free protease inhibitor cocktail [Roche] in RIPA buffer), scraped, collected, and spun at 15,000*g* for 30 min at 4°C. Supernatant was collected and incubated with immunoprecipitating antibody (p115 [C-9] [sc-74565] [Santa Cruz]) or phospho-PKC substrate (#2261, Cell Signaling) for 16–24 h at 4°C. The lysate-antibody mix was incubated with Protein A/G PLUS-Agarose beads (Santa Cruz), which were equilibrated in RIPA buffer beforehand, for 3 h. After four washes with ice-cold DPBS, bound protein was eluted by boiling for 10 min SDS sample buffer. Whole-cell lysates, supernatant, and immunoprecipitated samples were probed with antibodies to p115, phospho-PKC substrate, and β-tubulin (#2146S, Cell Signaling). Full gel blots can be found in S1 Raw Images.

For probing serine 240 on p115 as the PKC phosphorylation site, we expressed mCherry-tagged wild-type and S240A mutant p115. After p115 immunoprecipitation, phospho-PKC substrates were immunoblotted. Unfortunately, there was a nonspecific phospho-PKC substrate band with a size that coincided with mCherry-tagged p115 as it was seen when no plasmid was transfected. Thus, we swapped mCherry with a GFP and performed a GFP immunoprecipitation. However, the GFP pulldown was nonspecific. Therefore, we pulled down phospho-PKC substrates and probed for p115, which is what is shown in the paper.

### Rhotekin pulldown assay

RhoA activation was determined as described previously [47]. Briefly, cell lysates were incubated with the agarose-bound glutathione S-transferase-rhotekin-RhoA binding domain and then subjected to series of washes and centrifugations. Afterwards, 4 × Laemmli buffer was added and boiled for 5 min prior to SDS-PAGE analysis. Activated GTP-bound RhoA was detected by western blotting for RhoA (#2117S, Cell Signaling) and normalized to total RhoA in cell lysate.

### Time-lapse fluorescence imaging

Cells were washed twice with HBSS (Gibco) and subsequently imaged in HBSS in the dark at 37°C. Histamine (His; Sigma-Aldrich), pyrilamine (Pyr; Sigma-Aldrich), BAPTA (Cayman Chemical Company), EGTA (Sigma-Aldrich), ionomycin (Iono, LC Laboratories), Gö6983 (Calbiochem), PMA (Calbiochem), and CNO (Fisher) were added as indicated.

Epifluorescence imaging was performed on a Zeiss Axiovert 200M microscope (Carl Zeiss) equipped with a xenon lamp, a 40×/1.3 NA objective, and a cooled CCD controlled by METAFLUOR 7.7 software (Molecular Devices). For the Zeiss Axiovert 200M, the following excitation/emission filter combinations (maxima/bandwidths in nm) were used: BFP—EX380/10, EM475/25; CFP—EX420/20, EM475/25; YFP—EX495/10, EM535/25; RFP—EX568/55, EM653/95; CFP/YFP FRET—EX420/20, EM535/25. Exposure times were either 50 ms (for yellow channel), 100 ms (for red channel), or 500 ms (for all other channels), and images were acquired every 30 s. All epifluorescence experiments were subsequently analyzed using MetaFluor software. Pseudocolor images were generated in Image J.

### Time to half max analysis

MATLAB code was generated to computationally calculate t_1/2_ in an unbiased manner. Briefly, monophasic-responding curves were fit to the general exponential function:

$$A-Be^{-ct}$$

where *c* relates to t_1/2_ by the relationship:

$$\frac{\ln\left(2\right)}{c}$$

Biphasic-responding curves were fitted to a piece-wise exponential function. To divide the curve into two separate exponential functions, the second derivative was calculated and was fitted to a fourth order polynomial curve. The first local maximum of the curve fitted to the second derivative served as the time point for separating the first phase and second phase (*t*_*c*_). The timeframe for the first phase was defined as 0 < *t* < *t*_*c*_, and the timeframe for the second phase was defined as *t*_*c*_ < *t* < *t*_*max*_, where *t*_*max*_ is the time point corresponding to the max DORA RhoA emission ratio value post drug addition. The two phases were fitted to exponential curves similar to the analysis for the monophasic-responding curves. The end Y/C emission ratio (R) for the first peak (R_Peak 1_) was calculated by the respective emission ratio value for time point *t*_*c*_. An example biphasic curve fitted to two exponential curves based on our generated code is shown in S10 Fig. The code for the aforementioned analysis is available upon request.

### Fluorescence analysis

For biosensor analysis, raw fluorescence intensities were corrected by subtracting the background fluorescence intensity of a cell-free region from the emission intensities of biosensor-expressing cells. Y/C emission ratios (R) were then calculated at each time point. For calculating normalized Y/C emission ratio, the raw ratios were normalized by dividing the emission ratio at each time point by the starting ratio value at time zero (R_0_), which was defined as the emission ratio at the time point immediately preceding drug addition. For calculation of maximum Y/C emission ratio, the maximum changes from drug stimulation were reported for some of the bar graphs and were calculated as (R_max_ − R_0_)/R_0_, where R_max_ is the maximum emission ratio after the corresponding drug addition. For calculation of end R, the change in R after drug addition was reported for some of the bar graphs and were calculated as (end R_drug_−R_0_)/R_0_, where end R_drug_ is the emission ratio at the end of the imaging period after the corresponding drug addition. Residual RhoA activity was calculated as the emission ratio after pyrilamine addition R_Pyr_ over the ratio after histamine addition R_His_, R_Pyr_/R_His_. For biphasic responses, the peak percentage of total DORA RhoA response was calculated as the change in emission ratio from the indicated peak over the maximum ratio change, for peak 1 (R_peak 1_ –R_0_)/(max R–R_0_) and for peak 2 (R_peak 2_ –R_0_)/(max R–R_0_), where R_Peak 1_ is the end emission ratio for peak 1 and R_Peak 2_ is the end emission ratio for peak 2.

When using the RCaMP sensor [48], the normalized RFP intensity was calculated by dividing the raw RFP intensity at each time point by the starting RFP intensity at time zero (F/F_0_), which was defined as the RFP intensity at the time point immediately preceding drug addition. When using the ExRai CKAR sensor [30], the normalized excitation ratio was calculated by dividing the raw emission ratio (480 nm/400 nm) at each time point by the starting ratio value at time zero (R/R_0_), which was defined as the emission ratio at the time point immediately preceding drug addition.

For MRTF nuclear to cytosol analysis, the normalized nuclear/cytosol ratio was calculated by dividing the BFP intensity ratio between nucleus and cytosol (BFP_nucleus_/BFP_cytosol_) at each time point by the starting ratio value at time zero (N/N_0_), which was defined as the nuclear/cytosol ratio at the time point immediately preceding drug addition (N_0_). Residual MRTF was calculated as the nuclear/cytosol ratio after pyrilamine addition (N_Pyr_) over the ratio after histamine addition (N_His_) (N_Pyr_/N_His_). All graphs were plotted using GraphPad Prism 7 (GraphPad).

### Statistics and reproducibility

Statistical analyses were performed in GraphPad Prism 7 (GraphPad) and Microsoft Excel. All data were assessed for normality. Ordinary one-way ANOVA or two-way ANOVA tests were performed for data with multiple comparisons. For the rest of the statistics, normally distributed data pairwise comparisons were performed using unpaired two-tailed Student’s *t* test with Welch’s correction for unequal variances used as indicated. Statistical significance was set at *P* < 0.05. Average time courses shown in Figs 1A, 1D and 2A–2C (curves), 2E (curve), 3A, 3F, 4E, S1B-D, S1F-G, S2A-F, S2H-J (curve), S3, S4D (curve), S5B, and S6A-B are representative of at least three independently repeated experiments. Time courses shown in Figs 4A (curve), 4B (curve), S8, and S9 (curve) are single-cell traces that are representative of at least nine cells in total from three independently repeated experiments. Average time course from all experiments is shown in Fig 3F. Average bar graphs shown in Figs 1B, 1E, 1F, 2C–2E (bar), 3B, 4A (bar), 4B (bar) 4C, S1A, S1E, S2J, S2K (bar), S4B, S4D (bar), S5A, S6C, S7C and S9 (bar) depict combined data sets from at least three independent experiments.

### Computational modeling

All computational modeling was performed using Virtual Cell version 7.1.0.3. Briefly, the biphasic RhoA activation was modeled using either mass action kinetics for binding events or irreversible Michaelis-Mentin kinetics for enzyme-mediated reactions (S7A Fig). Although RhoGDIs were not considered experimentally in this paper, PKC is known to phosphorylate and thus deactivate RhoGDIs, and this PKC regulation of RhoGDIs was considered in the computational model [49,50]. All components were assumed to be homogenous and no spatial components were considered. All concentration and kinetic parameters were either attained from the literature or approximated as listed in S1 Table. Parameter approximation was done by inputting experimental data throughout the paper (including data from S6 Fig) into Virtual Cell’s parameter estimation module and fitted using the particle swarm COPASI method. The concentration for histamine and pyrilamine were those used in our experiments. Simulations were run using the IDA/CVODE method. A protocol where pyrilamine was added at indicated times was included in the simulations. The Virtual Cell BioModel “Histamine-RhoA Model_final” is available from the Public BioModels within the Virtual Cell software under the username “jzz002”. Virtual Cell can be downloaded from http://vcell.org [40,51].

We performed a sensitivity analysis to investigate how each parameter affected the kinetics and shape, but not magnitude, of the RhoA activity curve. Each parameter was individually perturbed upwards and downwards by one order of magnitude compared with the value in the original model. The RhoA activity curve was simulated and the ratio between active RhoA for each time point was calculated as:

$$\frac{active\;{RhoA}_{changed\;value}(t)}{active\;RhoA_{original\;value}(t)}$$

The standard deviation of these ratios was calculated and used as a measure of the effect of each perturbation. If the RhoA activity curve changed in magnitude but not in overall kinetics, this sensitivity metric was close to 0. If the overall kinetics of the RhoA activity curve changed, then the sensitivity metric will be higher. The sensitivity metric was calculated for the responses both to histamine (simulated as 100 μM histamine added at *t* = 0 and recorded for 20 min) and pyrilamine (simulated as 100 μM pyrilamine at *t* = 20 min post histamine addition, recorded for 30 min). The sensitivity metric for each parameter is reported in S1 Table. Parameters where one order magnitude changes in the original value resulted in sensitivity metric values for either histamine or pyrilamine response to be greater than 1 are highlighted in S7 Fig.

From the sensitivity analysis, the parameters that affected histamine-stimulated RhoA kinetics the most are either involved with H_1_HR/Gα_q_/p63 RhoGEF coupling or direct regulation of RhoA by p115 RhoGEF, GAP, and GDI (S1 Table and S7A Fig), consistent with the idea that direct effectors of RhoA have a key role in regulating RhoA activity dynamics. In addition, the results from the sensitivity analysis highlight key components (p63 and p115) of our mechanistic model, which proposes that histamine-stimulated biphasic activation of RhoA is due to p63-dependent and p115-dependent pathways.

## Supporting information

S1 Fig Activation of Gα_q_-coupled receptor induces biphasic activation of RhoA. (**A**) Average basal DORA RhoA emission ratio in HeLa cells with either nothing else coexpressed (red) or p190 RhoGAP (blue), p63 RhoGEF (orange), or p115 RhoGEF (purple) coexpression (nothing: *n* = 87 cells; +p190: *n* = 94 cells; +p63: *n* = 63 cells; +p115: *n* = 42 cells). ****P < 0.0001; ordinary one-way ANOVA followed by Dunnett’s multiple comparisons test (versus nothing transfected). (**B**) Representative average time courses ± SEM of the Y/C emission ratio changes in HeLa cells coexpressing p63 and DORA RhoA. Pyrilamine (100 μM) and then histamine (100 μM) was added to cells (*n* = 9 cells). (**C**) Representative average time courses ± SEM of the Y/C emission ratio changes in HeLa cells coexpressing p63 and RhoA1G. Histamine (100 μM) was added to cells (*n* = 8 cells). (**D**) Representative average time courses ± SEM of the Y/C emission ratio changes in MEF cells expressing DORA RhoA. Histamine (100 μM) was added to cells (*n* = 9 cells). (**E**) Quantification and representative western blot images of MEF cells simulated with 100 μM histamine. Numbers in the middle refer to minutes post histamine stimulation. For the Rhotekin pulldown samples, cell lysates were precipitated via beads covered with GST-tagged Rhotekin-RBD. Immunoblotting of RhoA of both the Rhotekin pulldown and whole-cell lysate samples show activation of RhoA in two waves from histamine stimulation (*n* = 3). Asterisks are statistics in comparison to 0 min: 0 min versus 2 min: *P = 0.047; 0 min versus 20 min: **P = 0.0063; ordinary one-way ANOVA followed by Dunnett’s multiple-comparisons test (versus 0 min). (**F**) Representative average time courses ± SEM of the Y/C emission ratio changes in HeLa cells coexpressing p63, DORA RhoA, and Gα_q_-DREADD. Cells were stimulated with 1 μM CNO (*n* = 6 cells). (**G**) Representative average time courses ± SEM of the Y/C emission ratio changes in MEF cells expressing DORA RhoA, p63, and p190. Histamine (100 μM) was added to cells (*n* = 18 cells). The underlying data for this figure can be found in S1 Data. CNO, Clozapine N-Oxide; MEF, mouse embryonic fibroblast; Y/C, yellow/cyan.
(TIF)

S2 Fig Delayed activation of RhoA is dependent on the Ca^2+^/PKC/p115 signaling axis. (**A-E**) Representative average time courses ± SEM of the Y/C emission ratio changes in HeLa cells coexpressing p63 and DORA RhoA. Cells were either stimulated with 100 μM histamine and then 5 min afterwards with 20 μM BAPTA (**A**) (*n* = 15 cells), imaged in HBSS imaging media containing 1 mM EGTA and then stimulated with 100 μM histamine (**B**) (*n* = 8 cells), stimulated with 1 μM ionomycin and then stimulated with 100 μM histamine (**C**) (*n* = 3 cells), stimulated with 100 μM histamine and then 5 min afterwards with 1 μM Gö6983 (*n* = 11 cells) (**D**), or stimulated with 50 ng/mL PMA and then stimulated with 100 μM histamine (**E**) (*n* = 3 cells). (**F**) Representative average time courses ± SEM of the Y/C emission ratio changes in HeLa cells expressing DORA RhoA and stimulated with 50 ng/mL PMA and then 1 μM Gö6983 (*n* = 5 cells). (**G**) Representative western blot images of p115 knockdown in HeLa cells. HeLa cells were transfected with either shRNA p115 (p115) or shRNA Scrambled (Sc) via calcium phosphate methods. Immunoblotting of p115 (top) shows substantial knockdown of p115 when transfecting shRNA p115. (**H, I**) Representative average time courses ± SEM of the Y/C emission ratio changes in HeLa cells transfected with DORA RhoA and either shRNA p115 (**H**) or shRNA Scrambled (**I**). Cells were stimulated with 100 μM histamine and then 100 μM pyrilamine (sh p115: *n* = 3 cells; sh Scrambled: *n* = 5 cells). (**J**) Left: Representative average time courses ± SEM of the Y/C emission ratio changes in HeLa cells expressing DORA RhoA, p63, and with p115 (red) or without p115 (blue) overexpressed and stimulated with 100 μM histamine (+p63 +p115: *n* = 7 cells; +p63: *n* = 14 cells). Right: Maximum emission ratio changes upon histamine stimulation (+p63 +p115: *n* = 22 cells; +p63: *n* = 27 cells). ****P < 0.0001; unpaired two-tailed Student’s *t* test. (**K**) HeLa cells expressing either p63 and p115 or p63 only were stimulated with 100 μM histamine. Percentage of total increase in DORA RhoA Y/C emission ratio contributed from the first phase (Peak 1%) or from the second phase (Peak 2%) (+p63 + p115: *n* = 22 cells; +p63: *n* = 27 cells). ****P < 0.0001; unpaired two-tailed Student’s *t* test. The underlying data for this figure can be found in S1 Data. BAPTA, 1,2-Bis(2-aminophenoxy)ethane-N,N,N′,N′-tetraacetic acid tetrakis (acetoxymethyl ester); EGTA, ethylene glycol-bis(β-aminoethyl ether)-N,N,N′,N′-tetraacetic acid; Gö6983, 3-[1-[3-(Dimethylamino)propyl]-5-methoxy-1H-indol-3-yl]-4-(1H-indol-3-yl)-1H-pyrrole-2,5-dione; HBSS, Hank’s balanced salt solution; PMA, phorbol myristate acetate; Y/C, yellow/cyan.
(TIF)

S3 Fig RhoA1G biosensor shows similar results to DORA RhoA sensor. (**A-B**) Representative average time courses ± SEM of the Y/C emission ratio changes in HeLa cells coexpressing p63 and RhoA1G. Cells were either pretreated with either 20 μM BAPTA (**A**) (*n* = 11 cells) or 1 μM Gö6983 (**B**) (*n* = 5 cells). Histamine (100 μM) was subsequently added to cells. The underlying data for this figure can be found in S1 Data. BAPTA, 1,2-Bis(2-aminophenoxy)ethane-N,N,N′,N′-tetraacetic acid tetrakis (acetoxymethyl ester); Gö6983, 3-[1-[3-(Dimethylamino)propyl]-5-methoxy-1H-indol-3-yl]-4-(1H-indol-3-yl)-1H-pyrrole-2,5-dione; Y/C, yellow/cyan.
(TIF)

S4 Fig PKC phosphorylates p115 RhoGEF on serine 240. (**A**) Representative western blot images of HeLa cells show that PKC phosphorylates p115. HeLa cells were either not stimulated, stimulated with 50 ng/mL PMA, or stimulated with 50 ng/mL PMA and 1 μM Gö6983. Afterwards, HeLa cell lysates were subjected to immunoprecipitation with antibodies to p115 and were immunoblotted for p115 (top) or phospho-PKC substrate (bottom). (**B**) Densitometry analysis of the immunoblot shown in (**A**) calculating the percentage of PKC-phosphorylated p115 over total p115. Average percentage ± SEM shown in bar graph among the various drug conditions (*n* = at least 4 for each condition). Nothing versus +PMA: *P = 0.017; +PMA versus +PMA + Gö6983: *P = 0.034; ordinary one-way ANOVA followed by Tukey’s multiple-comparisons test. (**C**) Domain structure of p115 RhoGEF [52]. Line indicates location of serine 240 residue. (**D**) Left: Representative average time courses ± SEM of the Y/C emission ratio changes in HeLa cells coexpressing DORA RhoA and either p115 WT (blue) or p115 S240A (red). PMA (50 ng/mL) was added to cells (p115 WT: *n* = 10 cells; p115 S240A: *n* = 4 cells). Right: Maximum emission ratio changes upon PMA and Gö6983 addition (p115 WT: *n* = 20 cells; p115 S240A: *n* = 11 cells). ****P < 0.0001; unpaired two-tailed Student’s *t* test. (**E**) Representative fluorescence images of HeLa cells transfected with p115 tagged with mCherry. Shown are images before drug addition, after addition of 100 μM histamine, and subsequent addition of 100 μM pyrilamine. Scale bar, 10 μm. The underlying data for this figure can be found in S1 Data. DH, Dbl homology domain; Gö6983, 3-[1-[3-(Dimethylamino)propyl]-5-methoxy-1H-indol-3-yl]-4-(1H-indol-3-yl)-1H-pyrrole-2,5-dione; PH, pleckstrin homology domain; PMA, phorbol myristate acetate; RH, RGS homology domain; WT, wild type; Y/C, yellow/cyan.
(TIF)

S5 Fig The Ca^2+^/PKC/p115 signaling axis enables persistent RhoA signaling. (**A**) HeLa cells expressing various proteins and treated with various drugs to increase or decrease flux into the Ca^2+^/PKC/p115 signaling axis. The Y/C emission ratio was measured after 100 μM histamine stimulation and then subsequently 100 μM pyrilamine addition (endogenous: *n* = 38 cells; +p63: *n* = 54 cells; +p63, BAPTA: *n* = 18 cells; +p63, -Extracellular Ca: *n* = 18 cells; +p63, Gö6983: *n* = 16 cells; +p115 S240A: *n* = 20 cells; +p63, ionomycin: *n* = 12 cells; +p63, PMA: *n* = 13 cells; +p115: *n* = 13 cells; +p63 +p115: *n* = 22 cells). Asterisks are in comparison to +p63, number signs are in comparison to endogenous condition: *P < 0.05, ***P < 0.001, ****P < 0.0001 two-way ANOVA followed by Dunnett’s multiple-comparisons test. ####*P* < 0.0001; unpaired two-tailed Student’s *t* test. (**B**) Representative average time courses ± SEM of the Y/C emission ratio changes in HeLa cells coexpressing p63 and DORA RhoA. Cells were stimulated with 100 μM histamine, 100 μM pyrilamine, and then 1 μM Gö6983 (*n* = 9 cells). The underlying data for this figure can be found in S1 Data. BAPTA, 1,2-Bis(2-aminophenoxy)ethane-N,N,N′,N′-tetraacetic acid tetrakis (acetoxymethyl ester); Gö6983, 3-[1-[3-(Dimethylamino)propyl]-5-methoxy-1H-indol-3-yl]-4-(1H-indol-3-yl)-1H-pyrrole-2,5-dione; PMA, phorbol myristate acetate; Y/C, yellow/cyan.
(TIF)

S6 Fig Calcium and PKC phosphorylation dynamics under histamine stimulation. (**A**, **B**) Representative average time courses ± SEM of the Y/C emission ratio changes in HeLa cells expressing either RCamp [48] (**A**) or ExRai CKAR [30] (**B**), to measure dynamics in calcium levels and PKC activity, respectively. HeLa cells were stimulated with either 100 μM histamine alone (**A**) or 100 μM histamine and then 100 μM pyrilamine (**B**) (RCamp: *n* = 8 cells; ExRai CKAR: *n* = 7 cells). This set of data was used to fit parameters in the computational model. (**C**) Left: Representative western blot images of HeLa cells simulated with either 100 μM histamine only or 100 μM histamine and then 100 μM pyrilamine. Numbers above refer to the number of minutes post histamine or pyrilamine addition. For all samples, cell lysates were immunoprecipitated with p115 antibody and immunoblotted for either p115 (top gel) or phospho-PKC substrates (bottom gel). Right: Densitometry analysis of the immunoblot shown on the left calculating the percentage of PKC-phosphorylated p115 over total p115. Average percentage ± SEM shown in bar graph amongst the various drug conditions (*n* = 6). Nothing versus 5 min histamine: *P = 0.047; Nothing versus 5 min histamine + 5 min pyrilamine: ***P = 0.0002; Nothing versus 5 min histamine + 20 min pyrilamine: **P = 0.0089; ordinary one-way ANOVA followed by Dunnett’s multiple-comparisons test (versus–His,–Pyr). The western blot results show prolonged PKC phosphorylation of p115 even after receptor antagonism. The underlying data for this figure can be found in S1 Data. Y/C, yellow/cyan.
(TIF)

S7 Fig Computational model of biphasic RhoA activation. (**A**) Construction of the computational model. Enzyme-mediated reactions are modeled with Michaelis-Menten kinetics (green). Binding events are modeled with mass action kinetics (black). Sensitivity analysis shows that the histamine response (blue), pyrilamine response (red), or both responses (purple) are sensitive (sensitivity metric > 1) to the highlighted parameters. (**B**) Computational model predictions for RhoA kinetics under various RhoGEF conditions. (**C**) Computational model aligns with experimental data. t_1/2_ comparison between computational predictions and experimental data (+p63, Phase 1 and Phase 2: *n* = 54 cells; +p63, Gö6983: *n* = 26 cells; +p115: *n* = 7 cells). The underlying data for this figure can be found in S1 Data. t_1/2_, time to half-maximal responses.
(TIF)

S8 Fig RhoA signaling kinetics direct MRTF-B nuclear translocation dynamics. (**A, B**) Individual cell traces of the DORA RhoA Y/C emission ratio changes (left axis) and the nuclear to cytosol ratio of MRTF-B (right axis) in HeLa cells expressing DORA RhoA, p63, and mTagBFP2-tagged MRTF-B. Cells were pretreated with Gö6983 to produce monophasic responders (**B**) with biphasic responders as controls (**A**). The underlying data for this figure can be found in S1 Data. Gö6983, 3-[1-[3-(Dimethylamino)propyl]-5-methoxy-1H-indol-3-yl]-4-(1H-indol-3-yl)-1H-pyrrole-2,5-dione; MRTF, myocardin-related transcription factor; Y/C, yellow/cyan.
(TIF)

S9 Fig RhoA signaling kinetics direct MRTF-A nuclear translocation dynamics. (**A, B**) Left: Individual cell traces of the DORA RhoA Y/C emission ratio changes (left axis) and the nuclear to cytosol ratio of MRTF-A (right axis) in HeLa cells expressing DORA RhoA, p63, and mTagBFP2-tagged MRTF-A. Cells were pretreated with Gö6983 to produce monophasic responders (**B**) with biphasic responders as controls (**A**). Right: residual RhoA activity and residual nuclearly localized MRTF-A for biphasic responders (**A**) and monophasic responders (**B**) (biphasic: *n* = 9 cells; monophasic: *n* = 10 cells). The underlying data for this figure can be found in S1 Data. Gö6983, 3-[1-[3-(Dimethylamino)propyl]-5-methoxy-1H-indol-3-yl]-4-(1H-indol-3-yl)-1H-pyrrole-2,5-dione; MRTF, myocardin-related transcription factor; Y/C, yellow/cyan.
(TIF)

S10 Fig Example time-to-half-maximum analysis for a biphasic curve. A representative biphasic curve that underwent time-to-half-maximum analysis. Two exponentials are fit for different time periods. The dividing point (*t*_*c*_) and its respective y-point on the curve, the two exponentials fitted to the separate phases, and the calculated t_1/2_ for each phase are depicted in the graph. t_1/2_, time to half-maximal responses.
(TIF)

S1 Raw Images Full gel images for Figs 2D, S1E, S2G, S4A and S6C. 2D: W = p115 WT, M = p115 S240A. S1E: numbers indicate minutes after histamine stimulation. S2G: p115 = shRNA p115, S = shRNA Scrambled, L = Lipofectamine, C = calcium phosphate. S6C: numbers indicate minutes post-histamine or post-pyrilamine addition (if both were added, histamine stimulation precedes pyrilamine addition). WT, wild type.
(TIF)

S1 Data (XLSX)

S1 Table Parameters used in computational model. (DOCX)

## Abbreviations

BAPTA: 1,2-Bis(2-aminophenoxy)ethane-N,N,N′,N′-tetraacetic acid tetrakis (acetoxymethyl ester)
CNO: Clozapine N-Oxide
DAG: diacylglycerol
EGTA: ethylene glycol-bis(β-aminoethyl ether)-N,N,N′,N′-tetraacetic acid
FRET: Förster resonance energy transfer
GAP: GTPase activating protein
GDI: guanine dissociation inhibitor
GEF: guanine nucleotide exchange factor
Gö6983: 3-[1-[3-(Dimethylamino)propyl]-5-methoxy-1H-indol-3-yl]-4-(1H-indol-3-yl)-1H-pyrrole-2,5-dione
H_1_HR: H_1_ histamine receptor
MEF: mouse embryonic fibroblast
MRTF-A/B: MRTF, myocardin-related transcription factor
PKN1: Protein Kinase N1
PMA: phorbol myristate acetate
RLU: relative light unit
SRF: Serum Response Factor
t_1/2_: time to half-maximal response
TNFα: tumor necrosis factor
Y/C: yellow/cyan
