# Supplementary figures and images for: Histamine-induced biphasic activation of RhoA allows for persistent RhoA signaling
Source: PLoS Biol. 2020 Sep 3;18(9):e3000866. doi: 10.1371/journal.pbio.3000866 (PMC7494096; doi:10.1371/journal.pbio.3000866)

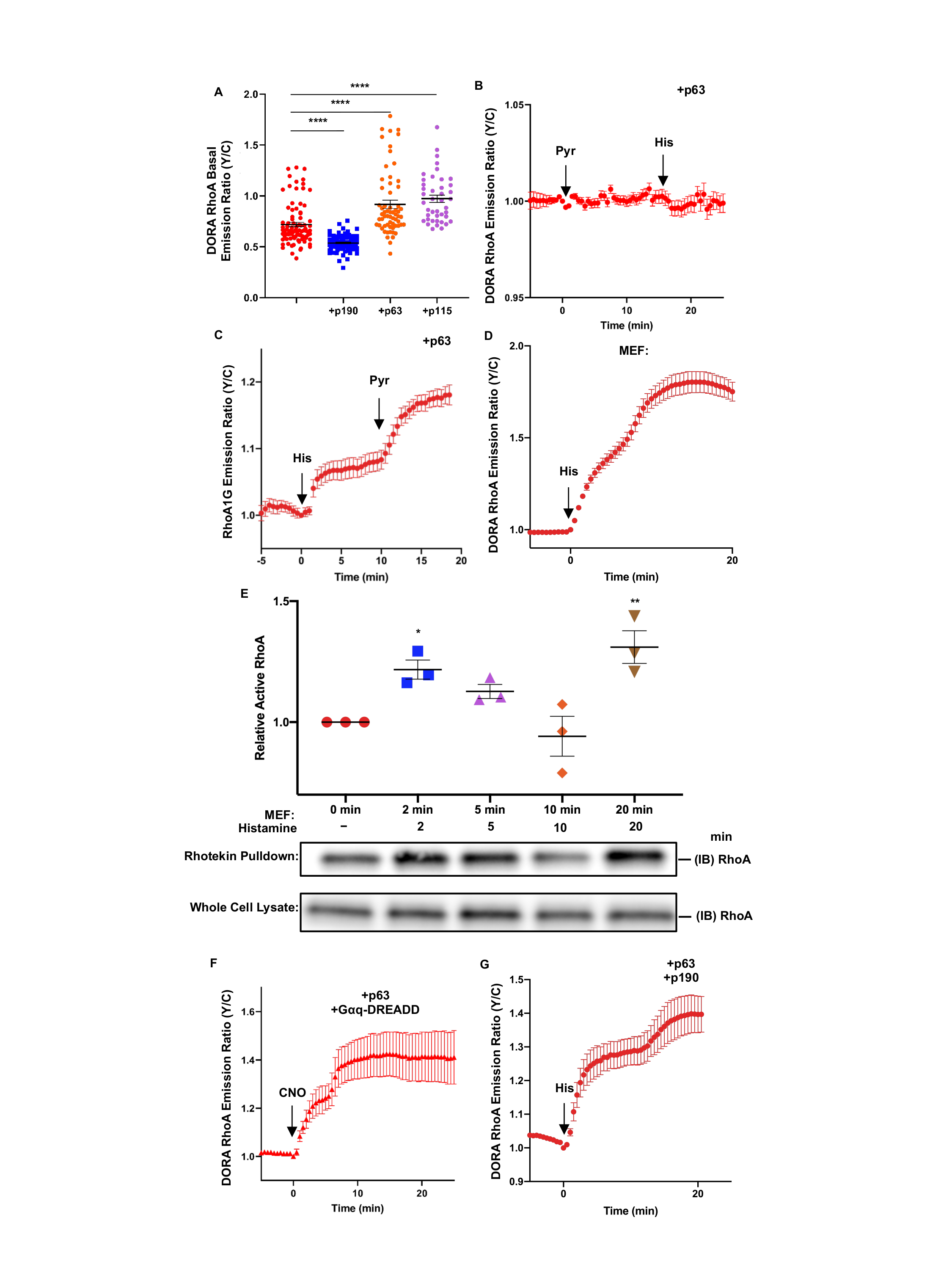

Supplement: S1 Fig — (A) Average basal DORA RhoA emission ratio in HeLa cells with either nothing else coexpressed (red) or p190 RhoGAP (blue), p63 RhoGEF (orange), or p115 RhoGEF (purple) coexpression (nothing: n = 87 cells; +p190: n = 94 cells; +p63: n = 63 cells; +p115: n = 42 cells). ****P < 0.0001; ordinary one-way ANOVA followed by Dunnett’s multiple comparisons test (versus nothing transfected). (B) Representative average time courses ± SEM of the Y/C emission ratio changes in HeLa cells coexpressing p63 and DORA RhoA. Pyrilamine (100 μM) and then histamine (100 μM) was added to cells (n = 9 cells). (C) Representative average time courses ± SEM of the Y/C emission ratio changes in HeLa cells coexpressing p63 and RhoA1G. Histamine (100 μM) was added to cells (n = 8 cells). (D) Representative average time courses ± SEM of the Y/C emission ratio changes in MEF cells expressing DORA RhoA. Histamine (100 μM) was added to cells (n = 9 cells). (E) Quantification and representative western blot images of MEF cells simulated with 100 μM histamine. Numbers in the middle refer to minutes post histamine stimulation. For the Rhotekin pulldown samples, cell lysates were precipitated via beads covered with GST-tagged Rhotekin-RBD. Immunoblotting of RhoA of both the Rhotekin pulldown and whole-cell lysate samples show activation of RhoA in two waves from histamine stimulation (n = 3). Asterisks are statistics in comparison to 0 min: 0 min versus 2 min: *P = 0.047; 0 min versus 20 min: **P = 0.0063; ordinary one-way ANOVA followed by Dunnett’s multiple-comparisons test (versus 0 min). (F) Representative average time courses ± SEM of the Y/C emission ratio changes in HeLa cells coexpressing p63, DORA RhoA, and Gαq-DREADD. Cells were stimulated with 1 μM CNO (n = 6 cells). (G) Representative average time courses ± SEM of the Y/C emission ratio changes in MEF cells expressing DORA RhoA, p63, and p190. Histamine (100 μM) was added to cells (n = 18 cells). The underlying data for this figure can be fo [file pbio.3000866.s001.tif]

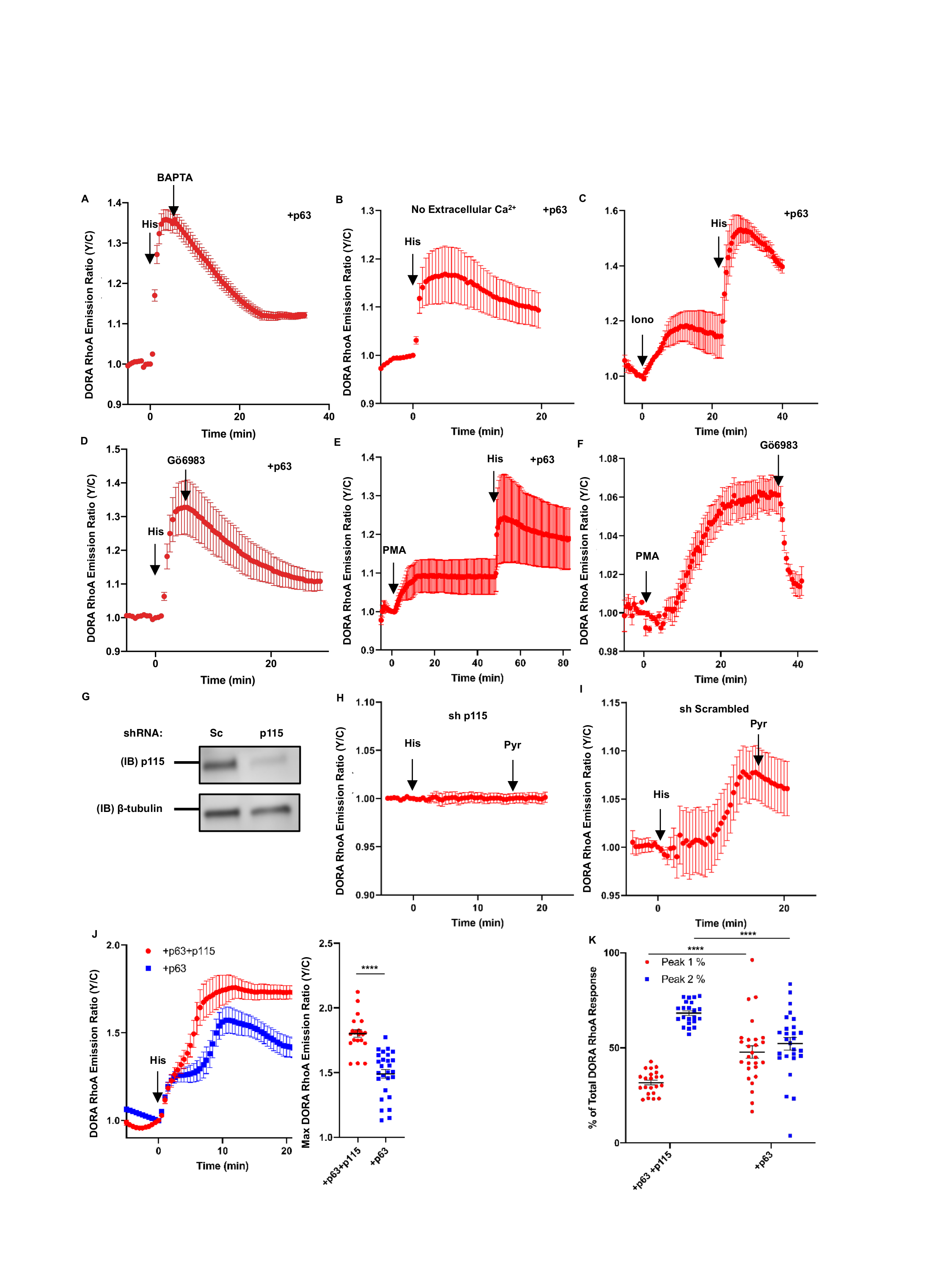

Supplement: S2 Fig — (A-E) Representative average time courses ± SEM of the Y/C emission ratio changes in HeLa cells coexpressing p63 and DORA RhoA. Cells were either stimulated with 100 μM histamine and then 5 min afterwards with 20 μM BAPTA (A) (n = 15 cells), imaged in HBSS imaging media containing 1 mM EGTA and then stimulated with 100 μM histamine (B) (n = 8 cells), stimulated with 1 μM ionomycin and then stimulated with 100 μM histamine (C) (n = 3 cells), stimulated with 100 μM histamine and then 5 min afterwards with 1 μM Gö6983 (n = 11 cells) (D), or stimulated with 50 ng/mL PMA and then stimulated with 100 μM histamine (E) (n = 3 cells). (F) Representative average time courses ± SEM of the Y/C emission ratio changes in HeLa cells expressing DORA RhoA and stimulated with 50 ng/mL PMA and then 1 μM Gö6983 (n = 5 cells). (G) Representative western blot images of p115 knockdown in HeLa cells. HeLa cells were transfected with either shRNA p115 (p115) or shRNA Scrambled (Sc) via calcium phosphate methods. Immunoblotting of p115 (top) shows substantial knockdown of p115 when transfecting shRNA p115. (H, I) Representative average time courses ± SEM of the Y/C emission ratio changes in HeLa cells transfected with DORA RhoA and either shRNA p115 (H) or shRNA Scrambled (I). Cells were stimulated with 100 μM histamine and then 100 μM pyrilamine (sh p115: n = 3 cells; sh Scrambled: n = 5 cells). (J) Left: Representative average time courses ± SEM of the Y/C emission ratio changes in HeLa cells expressing DORA RhoA, p63, and with p115 (red) or without p115 (blue) overexpressed and stimulated with 100 μM histamine (+p63 +p115: n = 7 cells; +p63: n = 14 cells). Right: Maximum emission ratio changes upon histamine stimulation (+p63 +p115: n = 22 cells; +p63: n = 27 cells). ****P < 0.0001; unpaired two-tailed Student’s t test. (K) HeLa cells expressing either p63 and p115 or p63 only were stimulated with 100 μM histamine. Percentage of total increase in DORA RhoA Y/C emission ratio contributed f [file pbio.3000866.s002.tif]

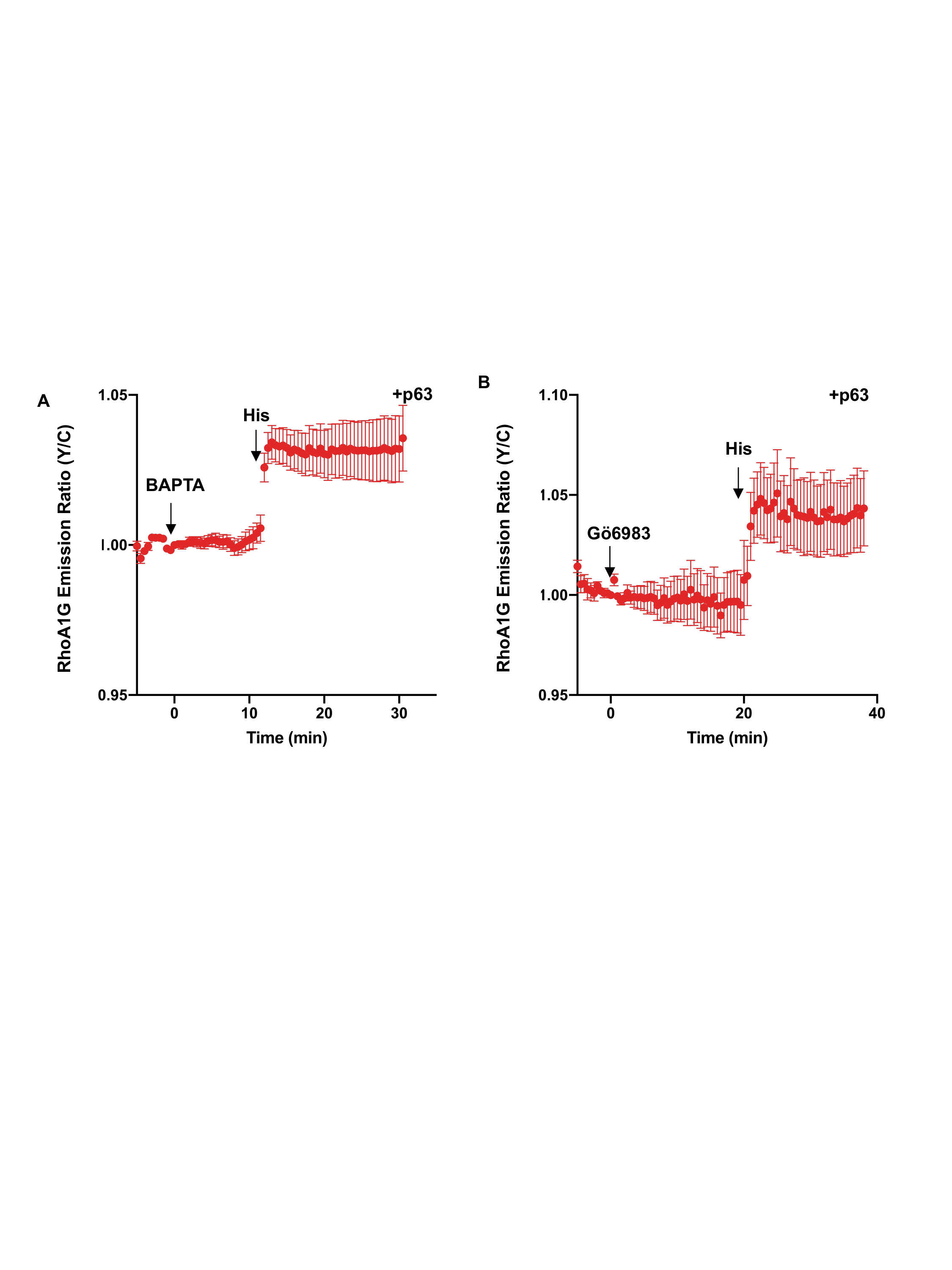

Supplement: S3 Fig — (A-B) Representative average time courses ± SEM of the Y/C emission ratio changes in HeLa cells coexpressing p63 and RhoA1G. Cells were either pretreated with either 20 μM BAPTA (A) (n = 11 cells) or 1 μM Gö6983 (B) (n = 5 cells). Histamine (100 μM) was subsequently added to cells. The underlying data for this figure can be found in S1 Data. BAPTA, 1,2-Bis(2-aminophenoxy)ethane-N,N,N′,N′-tetraacetic acid tetrakis (acetoxymethyl ester); Gö6983, 3-[1-[3-(Dimethylamino)propyl]-5-methoxy-1H-indol-3-yl]-4-(1H-indol-3-yl)-1H-pyrrole-2,5-dione; Y/C, yellow/cyan. (TIF) [file pbio.3000866.s003.tif]

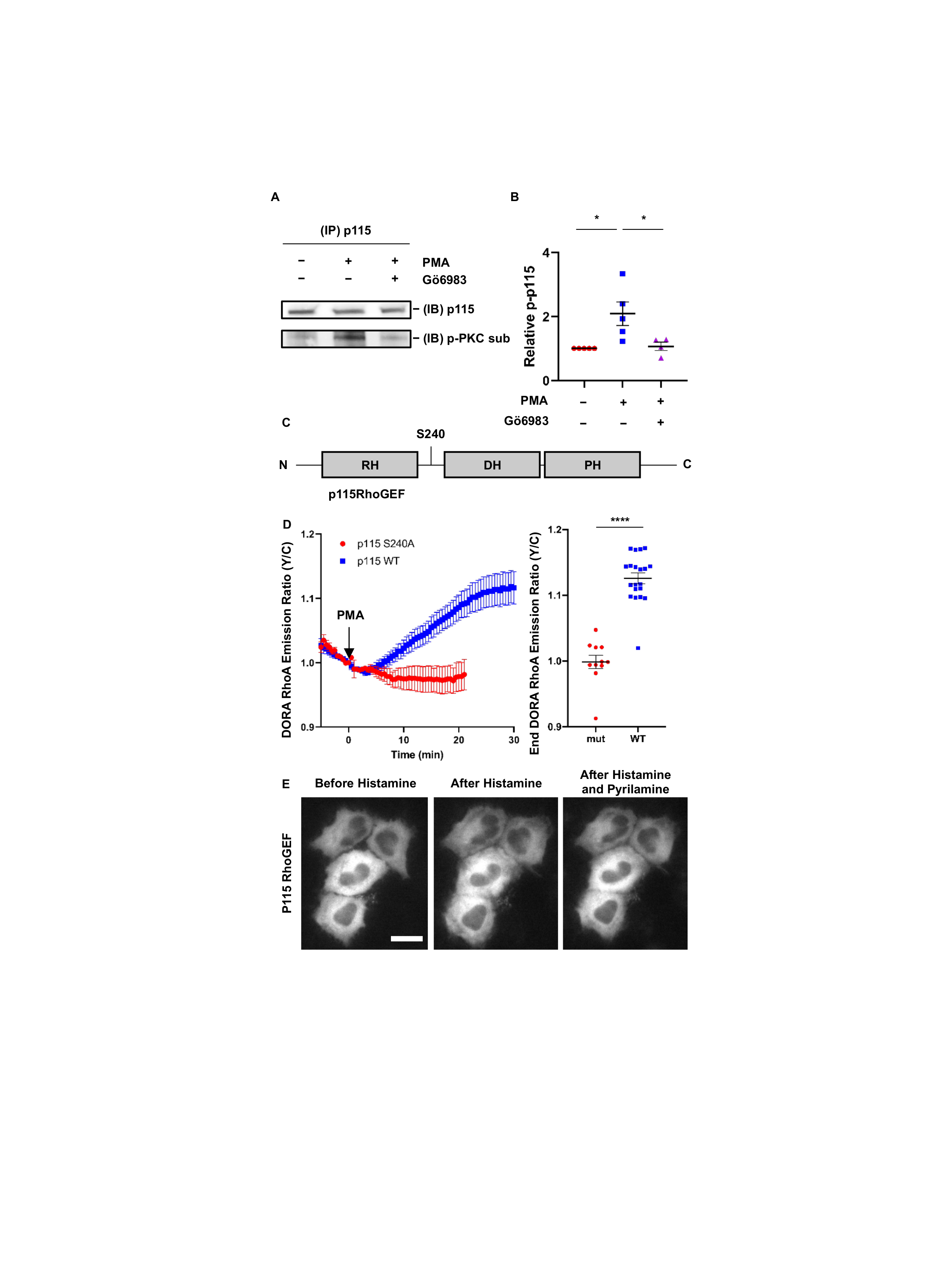

Supplement: S4 Fig — (A) Representative western blot images of HeLa cells show that PKC phosphorylates p115. HeLa cells were either not stimulated, stimulated with 50 ng/mL PMA, or stimulated with 50 ng/mL PMA and 1 μM Gö6983. Afterwards, HeLa cell lysates were subjected to immunoprecipitation with antibodies to p115 and were immunoblotted for p115 (top) or phospho-PKC substrate (bottom). (B) Densitometry analysis of the immunoblot shown in (A) calculating the percentage of PKC-phosphorylated p115 over total p115. Average percentage ± SEM shown in bar graph among the various drug conditions (n = at least 4 for each condition). Nothing versus +PMA: *P = 0.017; +PMA versus +PMA + Gö6983: *P = 0.034; ordinary one-way ANOVA followed by Tukey’s multiple-comparisons test. (C) Domain structure of p115 RhoGEF [52]. Line indicates location of serine 240 residue. (D) Left: Representative average time courses ± SEM of the Y/C emission ratio changes in HeLa cells coexpressing DORA RhoA and either p115 WT (blue) or p115 S240A (red). PMA (50 ng/mL) was added to cells (p115 WT: n = 10 cells; p115 S240A: n = 4 cells). Right: Maximum emission ratio changes upon PMA and Gö6983 addition (p115 WT: n = 20 cells; p115 S240A: n = 11 cells). ****P < 0.0001; unpaired two-tailed Student’s t test. (E) Representative fluorescence images of HeLa cells transfected with p115 tagged with mCherry. Shown are images before drug addition, after addition of 100 μM histamine, and subsequent addition of 100 μM pyrilamine. Scale bar, 10 μm. The underlying data for this figure can be found in S1 Data. DH, Dbl homology domain; Gö6983, 3-[1-[3-(Dimethylamino)propyl]-5-methoxy-1H-indol-3-yl]-4-(1H-indol-3-yl)-1H-pyrrole-2,5-dione; PH, pleckstrin homology domain; PMA, phorbol myristate acetate; RH, RGS homology domain; WT, wild type; Y/C, yellow/cyan. (TIF) [file pbio.3000866.s004.tif]

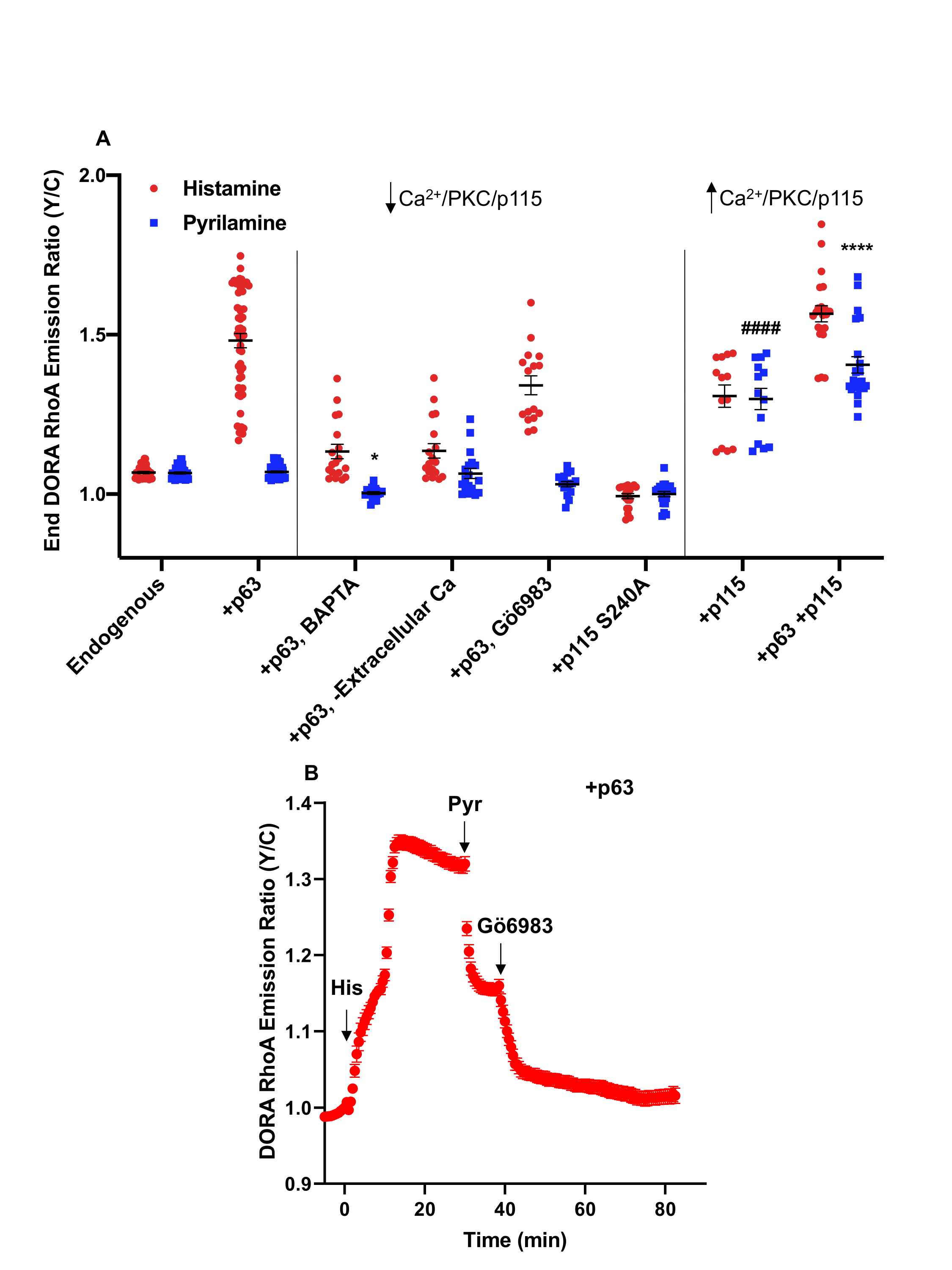

Supplement: S5 Fig — (A) HeLa cells expressing various proteins and treated with various drugs to increase or decrease flux into the Ca2+/PKC/p115 signaling axis. The Y/C emission ratio was measured after 100 μM histamine stimulation and then subsequently 100 μM pyrilamine addition (endogenous: n = 38 cells; +p63: n = 54 cells; +p63, BAPTA: n = 18 cells; +p63, -Extracellular Ca: n = 18 cells; +p63, Gö6983: n = 16 cells; +p115 S240A: n = 20 cells; +p63, ionomycin: n = 12 cells; +p63, PMA: n = 13 cells; +p115: n = 13 cells; +p63 +p115: n = 22 cells). Asterisks are in comparison to +p63, number signs are in comparison to endogenous condition: *P < 0.05, ***P < 0.001, ****P < 0.0001 two-way ANOVA followed by Dunnett’s multiple-comparisons test. ####P < 0.0001; unpaired two-tailed Student’s t test. (B) Representative average time courses ± SEM of the Y/C emission ratio changes in HeLa cells coexpressing p63 and DORA RhoA. Cells were stimulated with 100 μM histamine, 100 μM pyrilamine, and then 1 μM Gö6983 (n = 9 cells). The underlying data for this figure can be found in S1 Data. BAPTA, 1,2-Bis(2-aminophenoxy)ethane-N,N,N′,N′-tetraacetic acid tetrakis (acetoxymethyl ester); Gö6983, 3-[1-[3-(Dimethylamino)propyl]-5-methoxy-1H-indol-3-yl]-4-(1H-indol-3-yl)-1H-pyrrole-2,5-dione; PMA, phorbol myristate acetate; Y/C, yellow/cyan. (TIF) [file pbio.3000866.s005.tif]

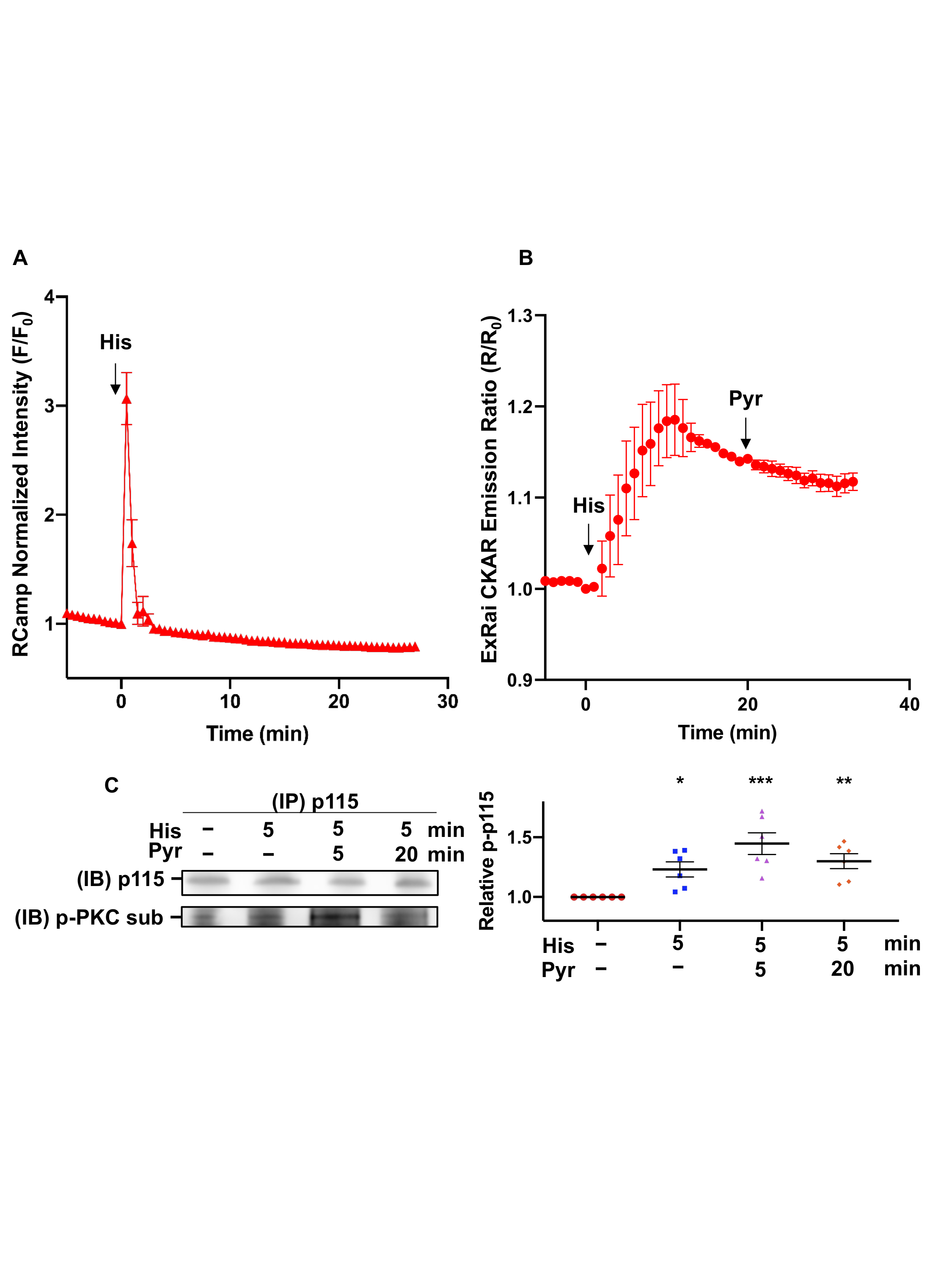

Supplement: S6 Fig — (A, B) Representative average time courses ± SEM of the Y/C emission ratio changes in HeLa cells expressing either RCamp [48] (A) or ExRai CKAR [30] (B), to measure dynamics in calcium levels and PKC activity, respectively. HeLa cells were stimulated with either 100 μM histamine alone (A) or 100 μM histamine and then 100 μM pyrilamine (B) (RCamp: n = 8 cells; ExRai CKAR: n = 7 cells). This set of data was used to fit parameters in the computational model. (C) Left: Representative western blot images of HeLa cells simulated with either 100 μM histamine only or 100 μM histamine and then 100 μM pyrilamine. Numbers above refer to the number of minutes post histamine or pyrilamine addition. For all samples, cell lysates were immunoprecipitated with p115 antibody and immunoblotted for either p115 (top gel) or phospho-PKC substrates (bottom gel). Right: Densitometry analysis of the immunoblot shown on the left calculating the percentage of PKC-phosphorylated p115 over total p115. Average percentage ± SEM shown in bar graph amongst the various drug conditions (n = 6). Nothing versus 5 min histamine: *P = 0.047; Nothing versus 5 min histamine + 5 min pyrilamine: ***P = 0.0002; Nothing versus 5 min histamine + 20 min pyrilamine: **P = 0.0089; ordinary one-way ANOVA followed by Dunnett’s multiple-comparisons test (versus–His,–Pyr). The western blot results show prolonged PKC phosphorylation of p115 even after receptor antagonism. The underlying data for this figure can be found in S1 Data. Y/C, yellow/cyan. (TIF) [file pbio.3000866.s006.tif]

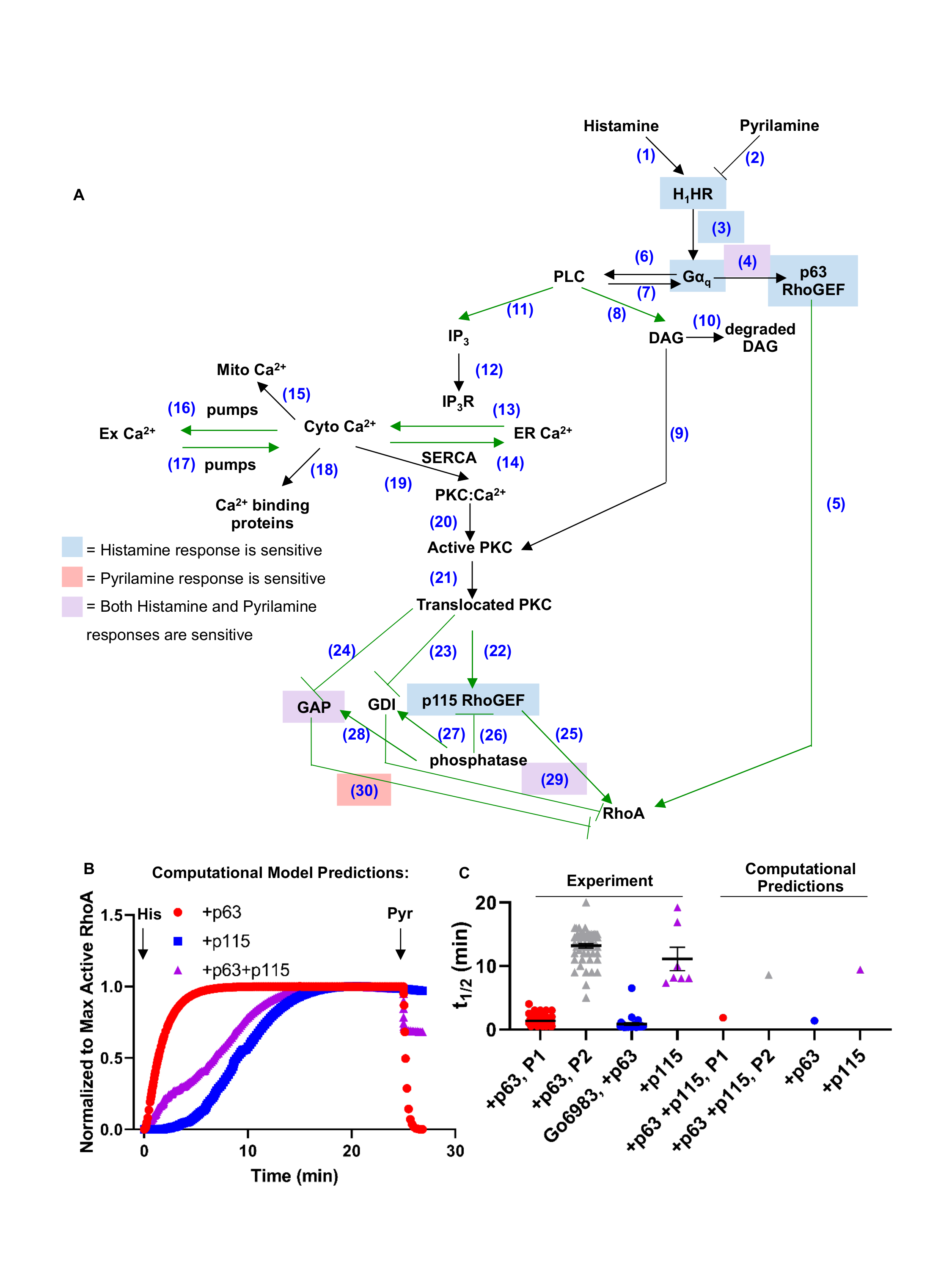

Supplement: S7 Fig — (A) Construction of the computational model. Enzyme-mediated reactions are modeled with Michaelis-Menten kinetics (green). Binding events are modeled with mass action kinetics (black). Sensitivity analysis shows that the histamine response (blue), pyrilamine response (red), or both responses (purple) are sensitive (sensitivity metric > 1) to the highlighted parameters. (B) Computational model predictions for RhoA kinetics under various RhoGEF conditions. (C) Computational model aligns with experimental data. t1/2 comparison between computational predictions and experimental data (+p63, Phase 1 and Phase 2: n = 54 cells; +p63, Gö6983: n = 26 cells; +p115: n = 7 cells). The underlying data for this figure can be found in S1 Data. t1/2, time to half-maximal responses. (TIF) [file pbio.3000866.s007.tif]

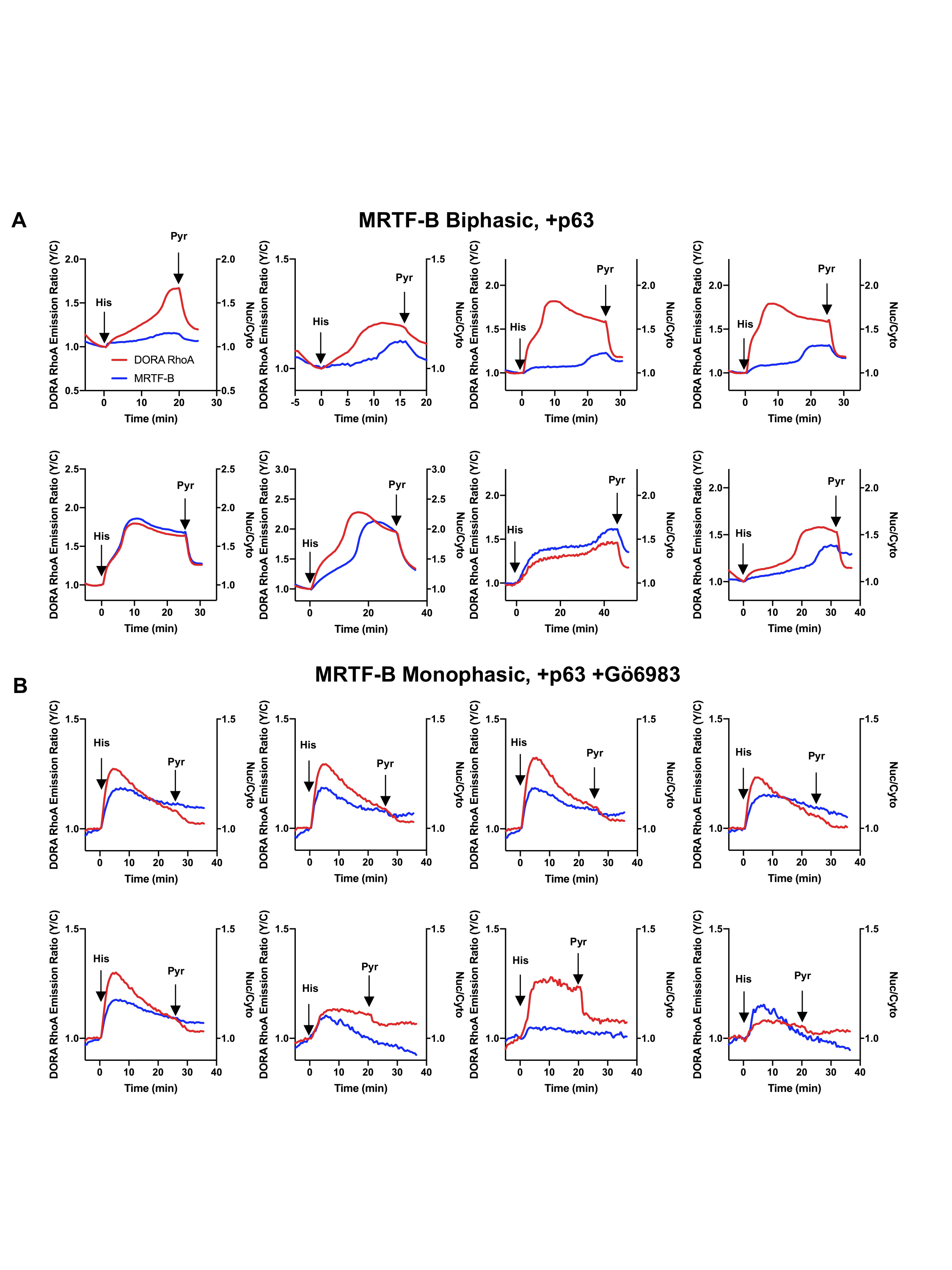

Supplement: S8 Fig — (A, B) Individual cell traces of the DORA RhoA Y/C emission ratio changes (left axis) and the nuclear to cytosol ratio of MRTF-B (right axis) in HeLa cells expressing DORA RhoA, p63, and mTagBFP2-tagged MRTF-B. Cells were pretreated with Gö6983 to produce monophasic responders (B) with biphasic responders as controls (A). The underlying data for this figure can be found in S1 Data. Gö6983, 3-[1-[3-(Dimethylamino)propyl]-5-methoxy-1H-indol-3-yl]-4-(1H-indol-3-yl)-1H-pyrrole-2,5-dione; MRTF, myocardin-related transcription factor; Y/C, yellow/cyan. (TIF) [file pbio.3000866.s008.tif]

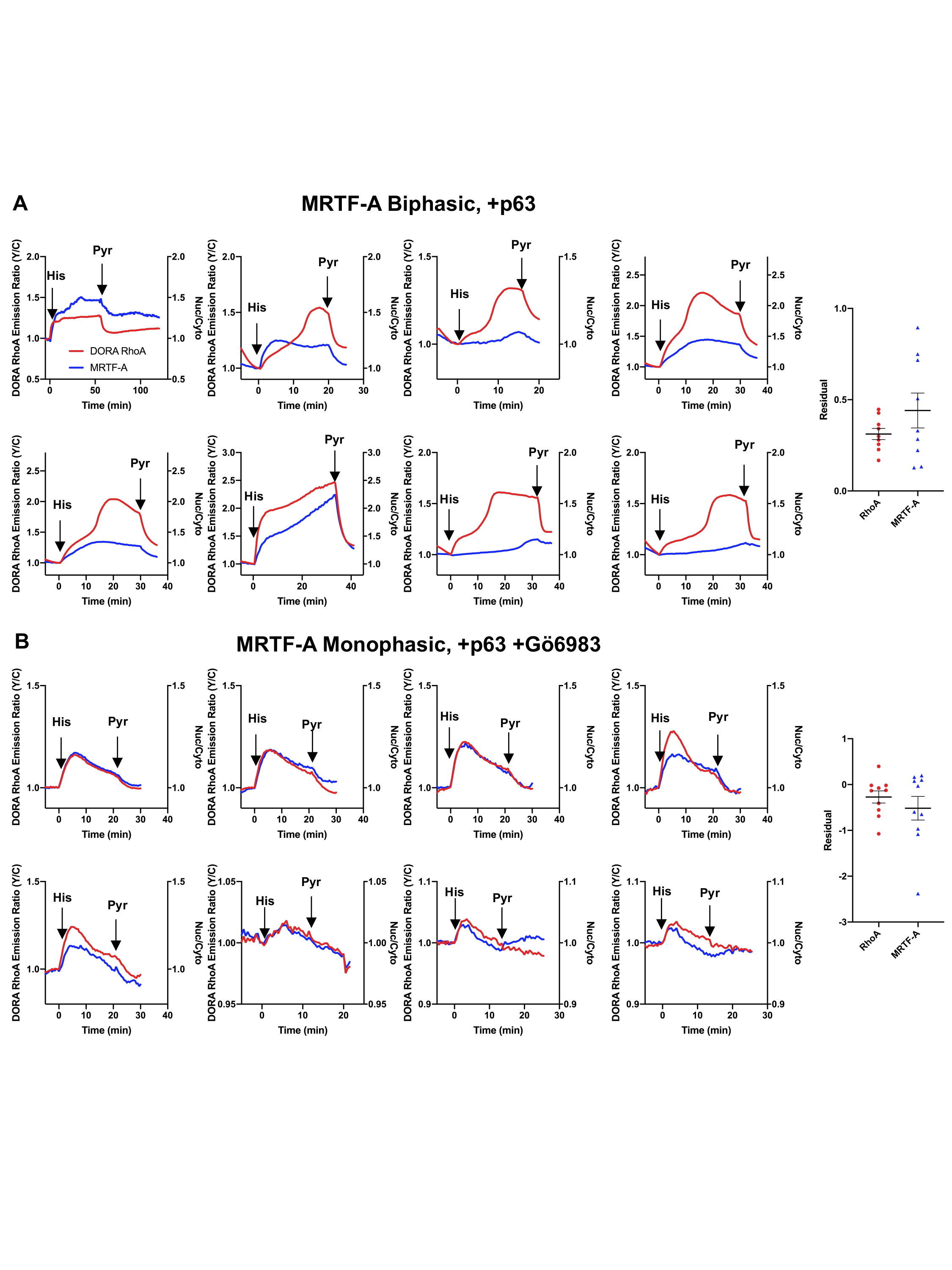

Supplement: S9 Fig — (A, B) Left: Individual cell traces of the DORA RhoA Y/C emission ratio changes (left axis) and the nuclear to cytosol ratio of MRTF-A (right axis) in HeLa cells expressing DORA RhoA, p63, and mTagBFP2-tagged MRTF-A. Cells were pretreated with Gö6983 to produce monophasic responders (B) with biphasic responders as controls (A). Right: residual RhoA activity and residual nuclearly localized MRTF-A for biphasic responders (A) and monophasic responders (B) (biphasic: n = 9 cells; monophasic: n = 10 cells). The underlying data for this figure can be found in S1 Data. Gö6983, 3-[1-[3-(Dimethylamino)propyl]-5-methoxy-1H-indol-3-yl]-4-(1H-indol-3-yl)-1H-pyrrole-2,5-dione; MRTF, myocardin-related transcription factor; Y/C, yellow/cyan. (TIF) [file pbio.3000866.s009.tif]

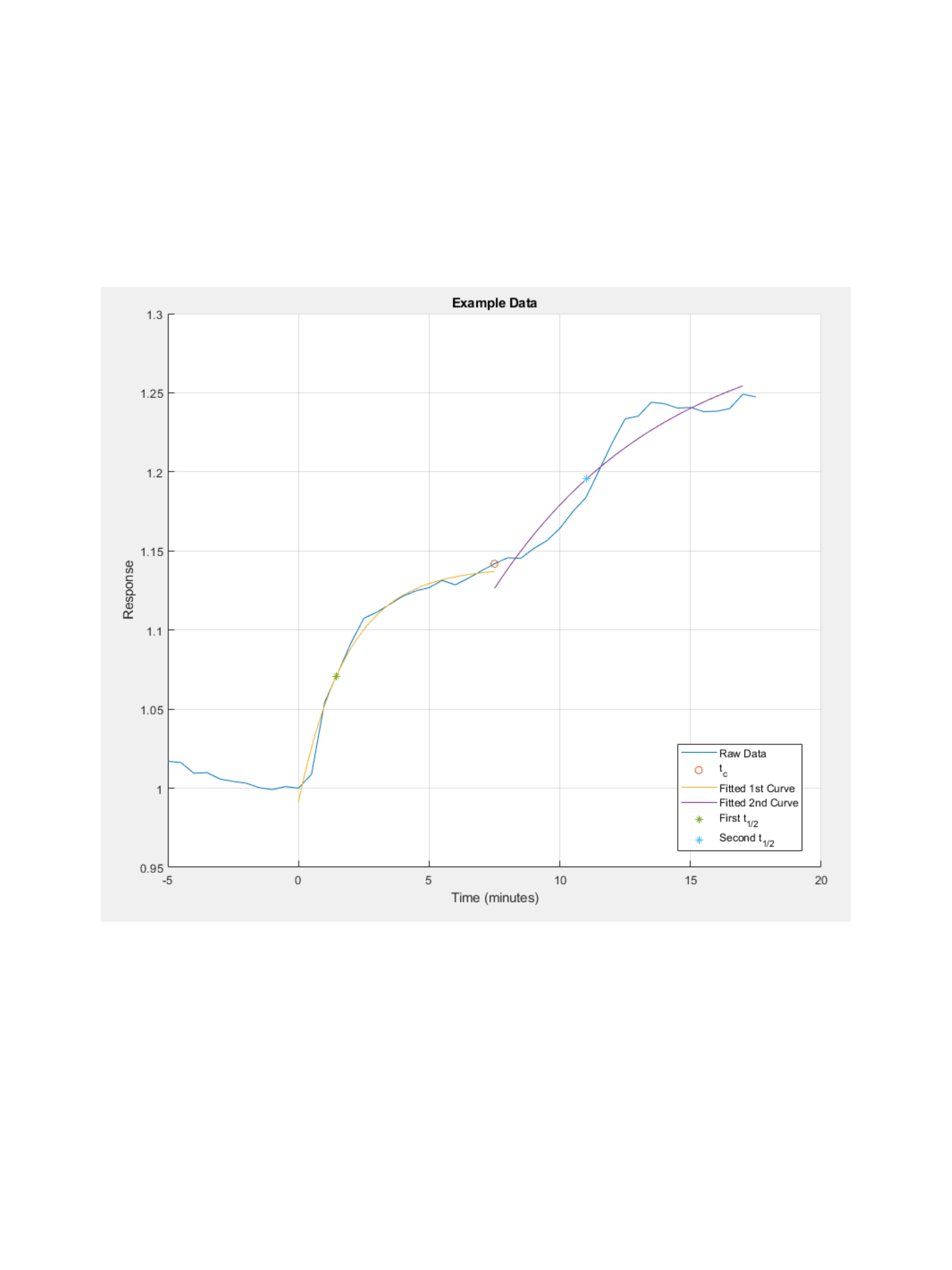

Supplement: S10 Fig — A representative biphasic curve that underwent time-to-half-maximum analysis. Two exponentials are fit for different time periods. The dividing point (tc) and its respective y-point on the curve, the two exponentials fitted to the separate phases, and the calculated t1/2 for each phase are depicted in the graph. t1/2, time to half-maximal responses. (TIF) [file pbio.3000866.s010.tif]

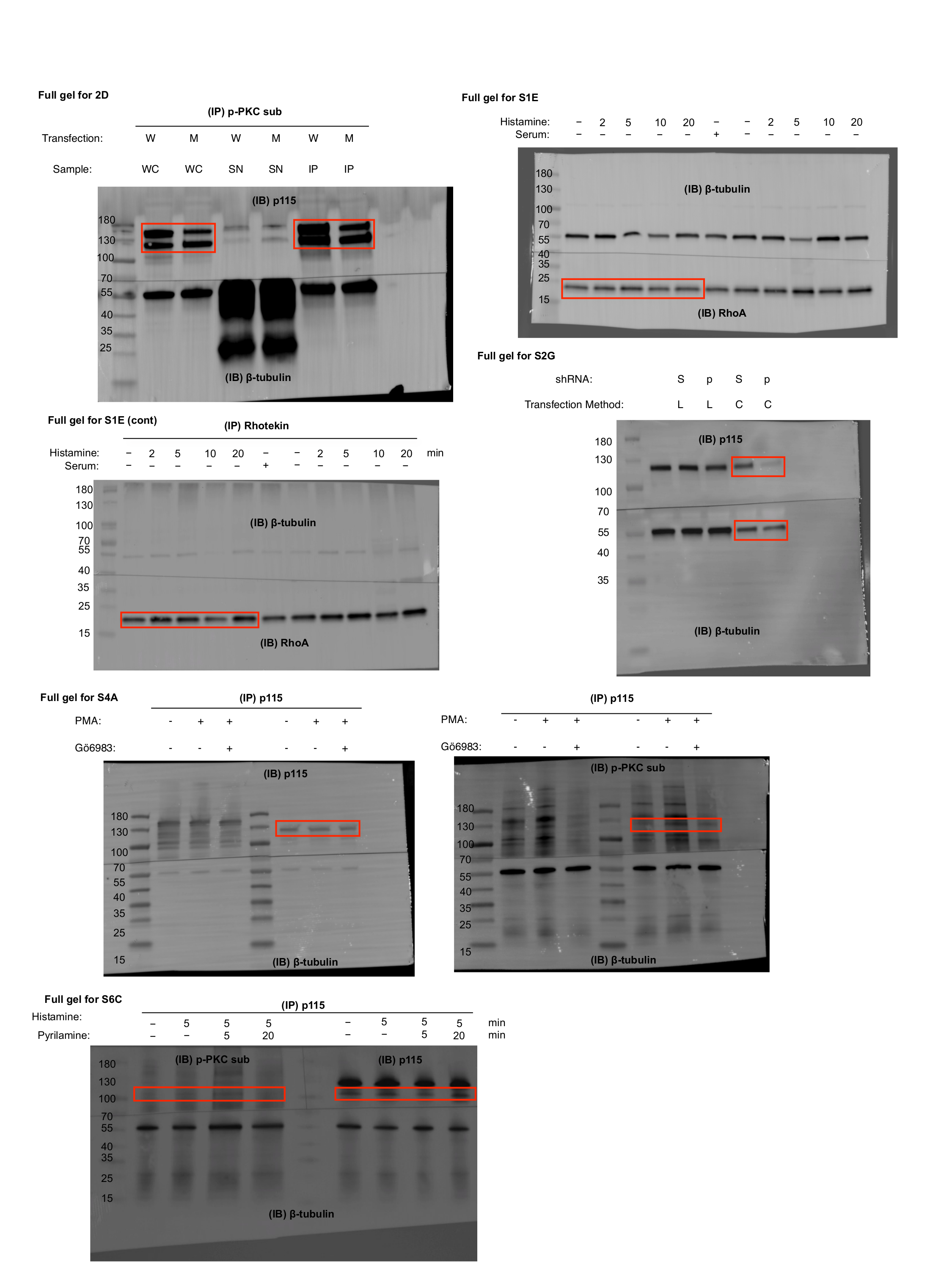

Supplement: S1 Raw Images — S1E: numbers indicate minutes after histamine stimulation. S2G: p115 = shRNA p115, S = shRNA Scrambled, L = Lipofectamine, C = calcium phosphate. S6C: numbers indicate minutes post-histamine or post-pyrilamine addition (if both were added, histamine stimulation precedes pyrilamine addition). WT, wild type. (TIF) [file pbio.3000866.s011.tif]
