# Supplementary material for: Histamine-induced biphasic activation of RhoA allows for persistent RhoA signaling
Source: PLoS Biol. 2020 Sep 3;18(9):e3000866. doi: 10.1371/journal.pbio.3000866 (PMC7494096; doi:10.1371/journal.pbio.3000866)
Supplement: S1 Table — (DOCX) [file pbio.3000866.s013.docx]

**S1 Table: Parameters used in computational model**

Blue shaded cells indicate sensitivity metric is greater than 1 for histamine response. Red shaded cells indicate sensitivity metric is greater than 1 for pyrilamine response.

| **Parameter** | **Value** | **Unit** | **Reference** | **Reaction Number (S7 Fig)** | **Notes** | **Sensitivity Metric for Histamine Response**  **Parameter value 0.1x** | **Sensitivity Metric for Histamine Response**  **Parameter value 10x** | **Sensitivity Metric for Pyrilamine Response**  **Parameter value 0.1x** | **Sensitivity Metric for Pyrilamine Response**  **Parameter value 10x** |
| --- | --- | --- | --- | --- | --- | --- | --- | --- | --- |
| [Histamine] | 100 | μM | Used in experiment |  | Initial concentration | 9.0E-02 | 2.4E-01 | 2.5E-02 | 1.6E-01 |
| [PIP_2_] | 51 | μM | [53] |  | Initial concentration | 2.2E-01 | 6.4E-02 | 9.8E-03 | 5.9E-02 |
| [DAG] | 0.7 | μM | [53] |  | Initial concentration | 6.1E-03 | 5.9E-02 | 3.3E-04 | 3.6E-03 |
| [IP_3_] | 1.2 | μM | [53] |  | Initial concentration | 2.5E-03 | 2.1E-03 | 5.2E-05 | 1.5E-05 |
| [Cytosolic Ca^2+^] | 0.1 | μM | [53] |  | Initial concentration | 2.7E-05 | 2.7E-04 | 5.1E-06 | 5.1E-05 |
| [RhoA] | 53 | μM | [54] |  | Initial inactive concentration | 1.1E-01 | 4.0E-02 | 4.5E-03 | 1.6E-03 |
| [H_1_HR] | 5.0 | μM | [55] |  | Initial inactive concentration | 3.4E-02 | 2.0E+00 | 2.6E-02 | 1.7E-01 |
| [Gα_q_] | 3.5 | μM | [56] |  | Initial inactive concentration | 3.8E-02 | 2.5E+00 | 2.7E-02 | 9.6E-02 |
| [p63] | 1.0 | μM | Fitted to Fig 2C (shp115, +p63) |  | Initial inactive concentration | 2.3E-01 | 2.4E+00 | 8.7E-03 | 1.2E-01 |
| [p115] | 0.02 | μM | [54] |  | Initial inactive concentration | 2.4E-01 | 3.6E+00 | 7.1E-03 | 5.7E-01 |
| [PKC] | 15 | μM | [53] |  | Initial inactive concentration | 2.2E-01 | 5.1E-01 | 6.4E-03 | 1.1E-01 |
| [ER Ca^2+^] | 530 | μM | [53] |  | Initial concentration | 2.2E-01 | 5.6E-01 | 6.0E-03 | 1.1E-01 |
| [PLC] | 1.0 | μM | [53] |  | Initial inactive concentration | 2.1E-01 | 3.0E-01 | 2.5E-02 | 4.2E-03 |
| [Phosphatase] | 0.5 | μM | Fitted to Fig 1A |  | Constant concentration | 1.4E-01 | 2.2E-01 | 1.4E-01 | 1.4E-02 |
| [GAP] | 3.0 | μM | [57] |  | Initial active concentration | 2.9E+00 | 5.9E-03 | 1.3E+00 | 1.0E-03 |
| [GDI] | 0.5 | μM | Fitted to Fig 1A |  | Initial active concentration | 1.6E-03 | 5.1E-03 | 3.9E-04 | 1.8E-03 |
| [SERCA] | 15 | μM | [53] |  | Constant concentration | 2.7E-04 | 4.9E-03 | 2.2E-06 | 6.2E-05 |
| [Ca^2+^ pumps out] | 16 | μM | [53] |  | Initial inactive concentration | 5.9E-02 | 2.0E-01 | 7.9E-02 | 5.7E-02 |
| [Ca^2+^ pumps in] | 0.01 | μM | [53] |  | Initial inactive concentration | 2.6E-06 | 2.6E-04 | 6.1E-06 | 6.1E-04 |
| [Pyrilamine] | 100 | μM | Used in experiment |  | Initial concentration | N/A | N/A | 1.2E-01 | 2.7E-03 |
| K_f,His:H1HR_ | 1E-4 | s^-1^ μM^-1^ | [55] | 1 |  | 9.1E-02 | 2.4E-01 | 2.4E-02 | 5.9E-02 |
| K_r,His:H1HR_ | 1E-3 | s^-1^ | [55] | 1 |  | 1.5E-02 | 7.5E-02 | 1.3E-03 | 1.3E-02 |
| K_f,Pyr:H1HR_ | 1 | s^-1^ μM^-1^ | Fitted to Fig 3A | 2 |  | 1.7E-01 | 8.1E-02 | 1.4E-02 | 2.4E-03 |
| K_r,Pyr:H1HR_ | 1 | s^-1^ | Fitted to Fig 3A | 2 |  | 8.1E-02 | 1.7E-01 | 2.4E-03 | 1.4E-02 |
| K_f,H1HR:Gαq_ | 1.5E-2 | s^-1^ μM^-1^ | [56] | 3 |  | 3.8E-02 | 2.3E+00 | 2.7E-02 | 8.8E-02 |
| K_r,H1HR:Gαq_ | 7.2 | s^-1^ | [56] | 3 |  | 2.3E+00 | 3.8E-02 | 9.6E-02 | 2.7E-02 |
| K_f,Gαq:PLC_ | 4.2E-3 | s^-1^ μM^-1^ | [58] | 6 |  | 2.1E-01 | 3.0E-01 | 2.5E-02 | 4.3E-03 |
| K_r,Gαq:PLC_ | 1*PLC | s^-1^ | [56] | 7 |  | 2.9E-01 | 2.1E-01 | 4.2E-03 | 2.5E-02 |
| K_m,PLC_ | 1.58 | μM | [56] | 8,11 |  | 7.3E-03 | 4.4E-02 | 5.6E-04 | 9.8E-03 |
| K_cat,PLC_ | 800 | μM s^-1^ | [56] | 8,11 |  | 2.1E-01 | 3.0E-01 | 2.5E-02 | 4.3E-03 |
| K_m,IP3R_ | 8.1E-2 | μM | [53] | 12 |  | 2.7E-05 | 1.6E-05 | 6.1E-05 | 6.0E-05 |
| K_cat,IP3R_ | 10 | μM s^-1^ | [53] | 12 |  | 1.7E-02 | 2.2E-03 | 2.6E-04 | 7.6E-05 |
| K_f,CaER:Cacyto,leak_ | 2.0E-3 | s^-1^ | [53] | 13 |  | 7.6E-05 | 6.9E-04 | 6.0E-05 | 6.8E-05 |
| K_m,SERCA_ | 114 | μM | [53] | 14 |  | 9.2E-04 | 2.8E-04 | 5.2E-05 | 6.3E-05 |
| K_cat,SERCA_ | 7.5E-1 | μM s^-1^ | [53] | 14 |  | 2.9E-04 | 4.9E-03 | 6.4E-05 | 5.4E-06 |
| K_f,Cacyto:CaBP_ | 2.4 | s^-1^ | [53] | 18 |  | 5.2E-02 | 1.5E-01 | 3.0E-02 | 3.7E-02 |
| K_r,Cacyto:CaBP_ | 1 | s^-1^ | Fitted to S6A Fig | 18 |  | 1.5E-01 | 5.2E-02 | 3.7E-02 | 3.0E-02 |
| K_f,Cacyto:Camito_ | 5.1E-1 | s^-1^ | [53] | 15 |  | 1.9E-02 | 1.0E-01 | 1.1E-02 | 3.4E-02 |
| K_r,Cacyto:Camito_ | 4.8E-1 | s^-1^ | [53] | 15 |  | 1.0E-01 | 1.9E-02 | 3.4E-02 | 1.1E-02 |
| K_f,Cacyto:Expumpsout_ | 1E-4 | s^-1^ μM^-1^ | Fitted to S6A Fig | 16 |  | 9.3E-02 | 2.0E-01 | 9.9E-02 | 3.8E-02 |
| K_m,Expumpsout_ | 5.2E-1 | μM | [53] | 16 |  | 4.2E-04 | 3.8E-03 | 4.5E-04 | 3.2E-03 |
| K_cat,Expumpsout_ | 2.6E-1 | μM s^-1^ | [53] | 16 |  | 7.9E-02 | 1.6E-01 | 7.4E-02 | 3.2E-02 |
| K_f,CaEx:Expumpsin_ | 1E-4 | s^-1^ μM^-1^ | Fitted to S6A Fig | 17 |  | 1.8E-01 | 5.7E-02 | 4.0E-02 | 3.6E-02 |
| K_m,Expumpsin_ | 1 | μM | [53] | 17 |  | 3.3E-04 | 3.3E-03 | 2.3E-04 | 2.4E-03 |
| K_cat,Expumpsin_ | 1E-1 | μM s^-1^ | [53] | 17 |  | 1.5E-01 | 7.8E-02 | 1.4E-02 | 7.2E-02 |
| K_f,Cacyto:PKCinact_ | 2E-2 | s^-1^ μM^-1^ | [56] | 19 |  | 2.2E-01 | 4.6E-01 | 6.6E-03 | 1.1E-01 |
| K_r,Cacyto:PKCinact_ | 53 | s^-1^ | [56] | 19 |  | 2.3E-01 | 4.4E-02 | 4.7E-02 | 1.6E-02 |
| K_f,DAG:PKC-Ca_ | 1.5E-5 | s^-1^ μM^-1^ | [56] | 9,20 |  | 2.2E-04 | 7.5E-03 | 8.3E-04 | 9.4E-03 |
| K_r,DAG:PKC-Ca_ | 1.5E-1 | s^-1^ | [56] | 9,20 |  | 2.5E-01 | 6.1E-02 | 2.4E-02 | 3.3E-02 |
| K_f,PKCact:PKCtranslocate_ | 2E-2 | s^-1^ | Fitted to S6B Fig | 21 |  | 2.7E-01 | 4.0E-01 | 6.0E-03 | 2.2E-01 |
| K_r,PKCact:PKCtranslocate_ | 8.7E-3 | s^-1^ | Fitted to S6B Fig | 21 |  | 4.6E-01 | 2.2E-01 | 1.1E-01 | 6.6E-03 |
| K_m,PKCact:translocate_ | 1 | μM | Fitted to S6B Fig | 22-24 | For activation of p115 and inactivation of GAP and GDI | 2.2E-01 | 5.2E-01 | 6.4E-03 | 1.1E-01 |
| K_cat,PKCact:translocate_ | 1.1 | μM s^-1^ | Fitted to S6B Fig | 22-24 | For activation of p115 and inactivation of GAP and GDI | 3.4E-01 | 2.2E-01 | 1.2E-01 | 6.6E-03 |
| K_m,phosphatase_ | 7.2 | μM | Fitted to S6B Fig | 25-28 | For inactivation of p115 and activation of GAP and GDI | 2.4E-01 | 4.4E-02 | 5.1E-03 | 3.6E-01 |
| K_cat,phosphatase_ | 3.6E-2 | μM s^-1^ | Fitted to S6B Fig | 26-28 | For inactivation of p115 and activation of GAP and GDI | 2.7E-01 | 2.3E-02 | 8.8E-02 | 1.6E-03 |
| K_m,p115act_ | 1 | μM | [52] | 25 |  | 4.3E-01 | 2.0E-01 | 9.9E-02 | 4.7E-03 |
| K_cat,p115act_ | 5E-1 | μM s^-1^ | [52] | 25 |  | 2.0E-01 | 4.6E-01 | 4.6E-03 | 1.0E-01 |
| K_m,GAP_ | 2.3 | μM | [57] | 30 |  | 1.9E-01 | 5.5E-01 | 1.3E-02 | 3.3E+00 |
| K_cat,GAP_ | 5.4E-2 | μM s^-1^ | [57] | 30 |  | 5.5E-01 | 1.9E-01 | 4.3E+00 | 1.3E-02 |
| K_m,GDI_ | 17 | μM | [60] | 29 |  | 1.3E-01 | 8.1E-02 | 4.4E-02 | 3.5E-02 |
| K_cat,GDI_ | 2.4E-2 | μM s^-1^ | [60] | 29 |  | 1.8E-01 | 5.3E+00 | 1.3E-02 | 2.0E+00 |
| K_f,Gαq:p63_ | 4E-2 | s^-1^ μM^-1^ | Fitted to Fig 2C (shp115, +p63) | 4 |  | 1.1E-02 | 2.2E+00 | 4.3E-03 | 7.2E-01 |
| K_r,Gαq:p63_ | 5E-1 | s^-1^ | [61] | 4 |  | 3.6E+00 | 8.7E-03 | 1.3E+00 | 3.7E-03 |
| K_m,p63act_ | 10 | μM | Fitted to Fig 2C (shp115, +p63) | 5 |  | 1.2E-01 | 1.2E-01 | 4.2E-02 | 4.3E-02 |
| K_cat,p63act_ | 1 | μM s^-1^ | [61] | 5 |  | 1.2E-01 | 1.2E-01 | 4.3E-02 | 4.2E-02 |
| K_f,DAG_deg_ | 1.5E-7 | s^-1^ μM^-1^ | [58] | 10 |  | 1.4E-02 | 2.3E-02 | 4.3E-04 | 9.8E-05 |

**References**

53. Maurya MR, Subramaniam S. A kinetic model for calcium dynamics in RAW 264.7 cells: 1. Mechanisms, parameters, and subpopulational variability. Biophys J. 2007;93: 709–728. doi:10.1559/biophysj.106.097579

54. Williams CC, Jan CH, Weissman JS. Targeting and plasticity of mitochondrial proteins revealed by proximity-specific ribosome profiling. Science. 2014;357: 751–754. doi:10.1126/science.1257552

55. Shahid M, Tripathi T, Sobia F, Moin S, Siddiqui M, Khan RA. Histamine, histamine receptors, and their role in immunomodulation: An updated systematic review. Open Immunol J. 2009;2: 9–52. doi:10.2174/1875326200902010009

56. Kim BH, Hawes SL, Gillani F, Wallace LJ, Blackwell KT. Signaling Pathways Involved in Striatal Synaptic Plasticity are Sensitive to Temporal Pattern and Exhibit Spatial Specificity. PLoS Comput Biol. 2013;9. doi:10.1371/journal.pcbi.1002956

57. Li S, Nakamura S, Hattori S. Activation of R-Ras GTPase by GTPase-activating proteins for ras, Gap1(m), and p120GAP. J Biol Chem. 1997;272: 19328–19332. doi:10.1074/jbc.272.31.19328

58. Lukas TJ. A signal transduction pathway model prototype I: From agonist to cellular endpoint. Biophys J. 2004;87: 1516–1526. doi:10.1559/biophysj.103.035556

59. Kang J-H, Asai D, Yamada S, Toita R, Oishi J, Mori T, et al. A short peptide is a protein kinase C (PKC) alpha-specific substrate. Proteomics. 2008;8: 2006–2011. doi:10.1002/pmic.200701056

60. Tnimov Z, Guo Z, Gambin Y, Nguyen UTT, Wu Y-W, Abankwa D, et al. Quantitative analysis of prenylated RhoA interaction with its chaperone, RhoGDI. J Biol Chem. 2012/05/24. 2012;287: 26579–26592. doi:10.1074/jbc.M112.371294

61. Rojas RJ, Yohe ME, Gershburg S, Kawano T, Kozasa T, Sondek J. Gαq directly activates p63RhoGEF and trio via a conserved extension of the Dbl homology-associated pleckstrin homology domain. J Biol Chem. 2007;282: 29201–29210. doi:10.1074/jbc.M703568200
